# Storage Stability and Functional Properties of Pomegranate Peel Extract-Enriched Vinegar Powders

**DOI:** 10.3390/plants15172630

**Published:** 2026-08-28

**Authors:** María de los Ángeles Martínez-Sánchez, Ginés Benito Martínez-Hernández, Antonio López-Gómez

**Affiliations:** 1 Food Safety and Refrigeration Engineering Group, Department of Agricultural Engineering, Universidad Politécnica de Cartagena, Paseo Alfonso XIII, 48, 30203 Cartagena, Spain; mariadelosangeles.martinez@edu.upct.es (M.d.l.Á.M.-S.); ginesbenito.martinez@upct.es (G.B.M.-H.); 2 Institute of Plant Biotechnology, Universidad Politécnica de Cartagena, Campus Muralla del Mar, Edificio I+D+I, 30202 Cartagena, Spain

**Keywords:** ultrasound-assisted extraction, agro-industrial byproducts, α-cyclodextrin, spray drying, phenolic compounds

## Abstract

Pomegranate peel is an agro-industrial byproduct rich in phenolic compounds with recognized functional properties. Its incorporation into stable food ingredients represents an attractive strategy for byproduct valorization. This study evaluated spray-dried vinegar powders enriched with pomegranate peel extract obtained by ultrasound-assisted extraction (UAE) using vinegar and α-cyclodextrin (αCD). Non-buffered and buffered formulations were compared with two commercial buffered powders during 6 months of storage at 22 °C. Morphological (particle size distribution and scanning electron microscopy images), physicochemical (pH, titratable acidity, moisture content, water activity and color), antioxidant (total phenolic content and DPPH radical scavenging activity), antimicrobial and sensory properties were evaluated. The powders maintained stable physicochemical properties, with moisture content remaining ≤1.2%, whereas commercial powders reached 3.0–3.9% after storage. Pomegranate-enriched powders showed total phenolic contents of 5.3–27.9 mg GAE/g_powder_ and antioxidant activity of 37.1–279.3 µmol TE/g_powder_, compared with 0.1–0.3 mg GAE/g_powder_ and 2.3–4.1 µmol TE/g_powder_ in the non-enriched formulations, while remaining stable throughout storage. Buffered enriched powders maintained in vitro antimicrobial activity against *Listeria monocytogenes*, whereas only non-buffered formulations inhibited *Salmonella enterica*. The powders were incorporated into a béchamel croquette formulation and showed preliminary sensory acceptability. These results support spray drying with αCD as an effective strategy for obtaining stable powdered ingredients from pomegranate peel byproducts under the evaluated conditions.

## 1. Introduction

Nowadays, the demand for sustainable and natural food ingredients with added functionality is increasing, together with the need to reduce food losses and promote the valorization of agri-food byproducts [1]. Current strategies such as recovery and purification of plant byproducts have emerged to convert agricultural waste into value-added ingredients while contributing to more sustainable food production [2,3]. Among these byproducts, pomegranate peel (*Punica granatum* L.) has attracted particular attention because it represents one of the richest sources of phenolic compounds, including ellagitannins (e.g., punicalagin and punicalin), hydroxybenzoic acids (e.g., gallic acid), ellagic acid, flavan-3-ols (e.g., catechin), anthocyanins, and hydroxycinnamic acids, which have been widely associated with antioxidant, antimicrobial, and immunomodulatory activities [4,5]. Besides their recognized health-promoting properties, pomegranate-derived products are widely consumed as fresh fruit or processed into juices, jams, and dietary supplements, making pomegranate peel an attractive raw material for the development of new plant-derived functional ingredients [6,7].

As phenolic compounds are particularly susceptible to degradation during processing and storage, preserving their stability remains one of the main challenges for their incorporation into food ingredients and products [8]. In recent years, ultrasound-assisted extraction (UAE) has emerged as an efficient green technology for recovering bioactive compounds, improving mass transfer into the solvent while reducing extraction time and solvent consumption compared with conventional extraction methods [9,10]. Nevertheless, one of the main obstacles limiting the application of pomegranate-derived phenolic extracts in the food industry is the widespread use of organic solvents during extraction, which restricts their direct incorporation into food formulations [11].

In a previous study, our research group developed an α-cyclodextrin (αCD)-assisted UAE process using vinegar as a food-grade extraction medium to recover phenolic compounds from pomegranate peel [12]. Distilled vinegar is widely used in food industry because of its high acetic acid content, responsible for both preservative functionality and its typical pungent flavor [13,14]. Because of its acidic and sour nature, the use of conventional vinegar faces some limitations when added to some food products [15,16]. Buffered vinegar has therefore developed as an interesting alternative, since partial neutralization with potassium or sodium salts reduces sourness while maintaining the antimicrobial properties of vinegar and shows potential to contribute to salt perception [17,18,19].

Although the extraction of phenolic compounds is an essential first step in the valorization of agri-food byproducts, preserving their stability after processing is just as important if these extracts are intended to be incorporated into food products. In this context, the microencapsulation of pomegranate peel phenolic compounds within a vinegar matrix could not only protect bioactive compounds against quality and bioactivity losses but also produce new plant-derived powdered ingredients free from chemical preservatives [20]. Spray drying is a simple, fast and economic encapsulation technique to convert liquid extracts into stable powders, while protecting sensitive compounds from environmental conditions by enclosing them inside a wall material [21]. It has been successfully applied in the food industry for the entrapment of polyphenol-rich extracts, reducing quality losses and facilitating their incorporation into different food matrices [22]. While maltodextrin (MD) is one of the most used carrier agents for the spray-drying of fruit-derived products and vinegar formulations [20,23], cyclodextrins (CD) have also demonstrated a high capacity to protect sensitive bioactive compounds during spray drying, particularly in pomegranate-derived ingredients [24]. Although the use of αCD as wall material has been less extensively studied than βCD or γCD, it is recognized as dietary fiber suitable for food applications and represents a promising carrier for phenolic-enriched formulations [25,26]. However, the evaluation of storage stability of spray-dried pomegranate peel-enriched vinegar as well as the influence of vinegar buffering on the technological properties of these powdered formulations has not yet been studied.

Thus, the aim of this experimental work was to develop an innovative spray-dried pomegranate peel extract-enriched vinegar formulation obtained by the UAE system previously developed by our research group. To this end, the storage stability of non-enriched and pomegranate peel-extract-enriched, both non-buffered and buffered, vinegar formulations was evaluated for up to 6 months at 22 °C. Morphological, physicochemical, antioxidant, antimicrobial and sensory characteristics were studied, in comparison to two commercial non-enriched buffered vinegar powders as references. In addition, the developed powders were incorporated into a model breaded product to assess the sensory acceptance of the pomegranate peel extract-enriched formulations as functional food ingredients.

## 2. Results and Discussion

### 2.1. Morphological Characterization

#### 2.1.1. Particle Size Distribution

Table 1 presents the particle size distribution of the studied formulations during 6 months of storage. At month 0, the formulations developed in this study showed considerably smaller particle sizes than the commercial buffered references. D_50_ values ranged from 26.1–59.8 µm for the developed formulations, whereas B-C1 and B-C2 showed D_50_ values of 480.5 ± 0.1 and 109.6 ± 0.1 µm, respectively. These differences most likely reflect the different formulation strategies, encapsulating agents and spray-drying conditions employed during manufacture. Previous studies have reported that more compact spray-dried particles are produced when αCD is used as encapsulating carrier [25,27], which may have contributed to the comparatively small particle sizes obtained for the formulations developed in the present study.

N-PV showed lower D_10_ and D_50_ values (4.2 ± 0.1 and 26.1 ± 0.1 µm, respectively) than N-NV (6.9 ± 0.1 and 59.8 ± 0.1 µm, respectively; *p* < 0.05) but also exhibited a substantially broader particle size distribution, as reflected by its higher D_90_ and Span values. Similar behaviour has been described for spray-dried powders containing plant-derived polyphenol-rich extracts, where the incorporation of plant material modifies the characteristics of the feed and results in more heterogeneous particle populations after atomization [5,11,28]. Likewise, potassium buffering broadened the particle size distribution of the developed powders compared with N-NV. Nevertheless, all the experimental formulations presented D_50_ values below 60 µm, remaining within the particle size range commonly reported for spray-dried food powders [21,28].

During storage, although most formulations maintained relatively stable particle size characteristics, N-PV showed significant decreases in D_10_, D_50_, D_90_ and Span values after 6 months (*p* < 0.05), resulting in a more homogeneous particle size distribution. These results may be consistent with a gradual rearrangement of the powder structure during storage, although this interpretation remains hypothetical because the specific structural changes and the mechanisms underlying this behaviour were not directly investigated in the present study. In contrast, the limited changes observed in particle size values in B-PV, although significant (*p* < 0.05), indicated good physical stability under the evaluated conditions. The commercial powders also remained relatively stable, with slight but significant differences at the end of the storage time.

Nevertheless, although the incorporation of pomegranate peel extract and potassium buffering influenced the initial (month 0) particle size distribution of the spray-dried powders compared to non-enriched powders, it did not compromise their physical stability throughout storage. The absence of major alterations in the particle distribution was consistent with good physical stability of the powders under the evaluated storage conditions and suggests limited changes in their particle size characteristics during storage [21].

#### 2.1.2. Surface Morphology

**Figure 1:**
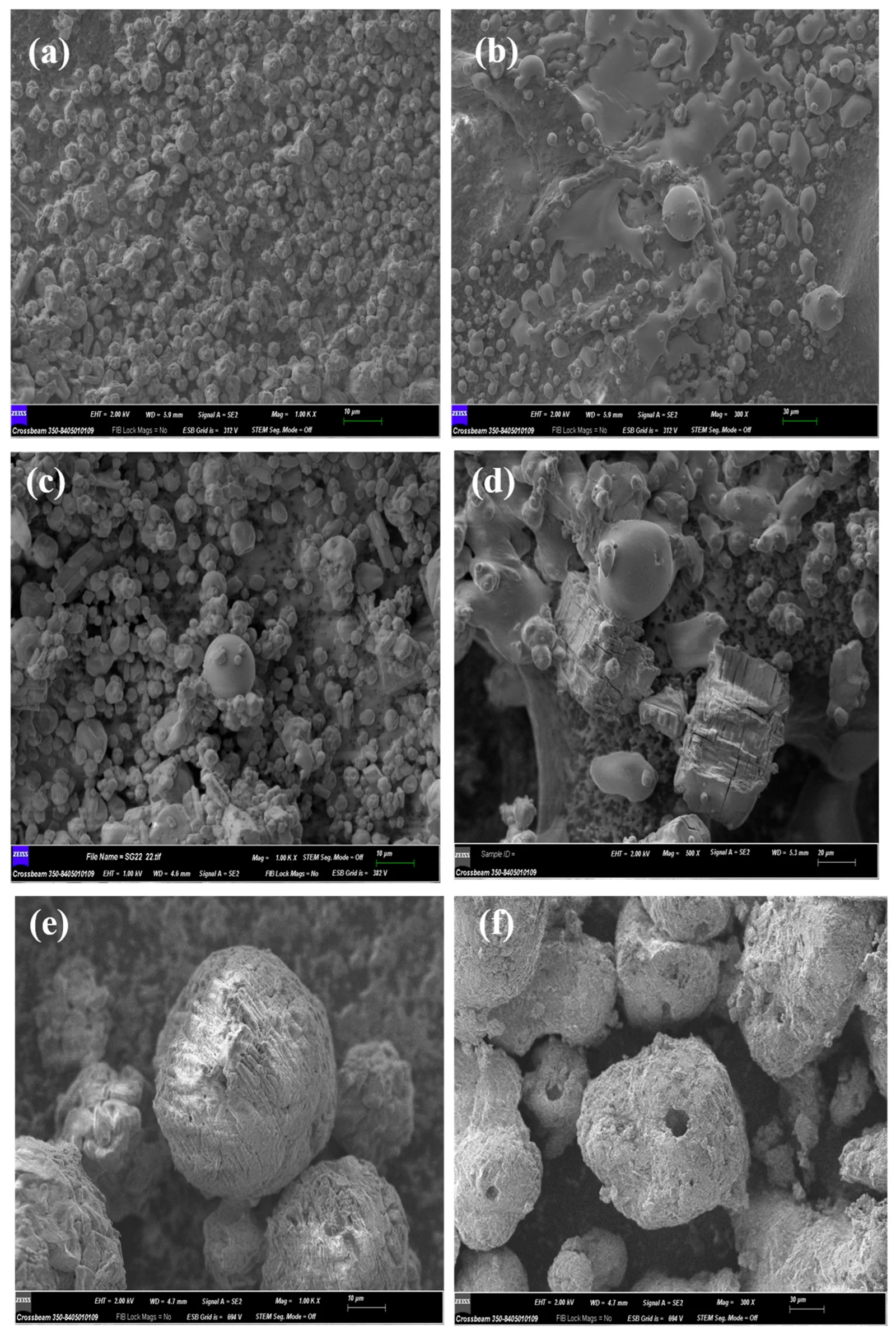
SEM micrographs of non-enriched (**a**,**b**): N-NV ((**a**), 1000×) and B-NV ((**b**), 300×); pomegranate peel extract-enriched (**c**,**d**): N-PV ((**c**), 1000×), B-PV ((**d**), 500×); and commercial buffered references (**e**,**f**): B-C1 ((**e**), 1000×) and B-C2 ((**f**), 300×) at the beginning of storage time (month 0).

The resulting effect of spray drying on the morphological structure of the non-enriched and pomegranate peel extract-enriched formulations was investigated by SEM analysis, in comparison to commercial vinegar powders (Figure 1). The morphology of the particles was influenced by the atomization conditions and the use of αCD as an encapsulation agent in the formation of the resulting microcapsules [27]. A heterogeneous diversity was observed in the structure and size of the microcapsules, in accordance with the particle size distribution obtained in this study (Table 1), suggesting a direct relationship between these parameters [28]. In general, spray-dried non-enriched and pomegranate peel extract-enriched particles showed flat surfaces with spherical shapes with no structural collapse (Figure 1a–d), indicative of an efficient spray drying process [29]. No major differences were found in particles’ shape when observing non-enriched (N-NV and B-NV) and pomegranate peel extract-enriched formulations (N-PV and B-PV), confirming a well-adjusted drying process for the plant-derived formulations investigated. Those smooth surfaces with no damage or concavities suggested adequate encapsulation of the liquid extracts, usually associated with adequate formulation and process control that favors stability of the resulting powders [30,31]. Previous studies also reported rounded-shape particles in microencapsulated pomegranate juice spray-dried with maltodextrin and gum arabic as encapsulating materials, with a smooth-to-wrinkled surface dependent on the drying conditions and on particle size [11]. In accordance with these studies, the morphology of the commercial references differed from rounded shapes as larger particle sizes were registered, compared with the developed formulations. Similarly, it has also been described mostly spherical in shape particles of spray-dried β-glucan powders with maltodextrin from *Pleurotus ostreatus* [32].

**Figure 2:**
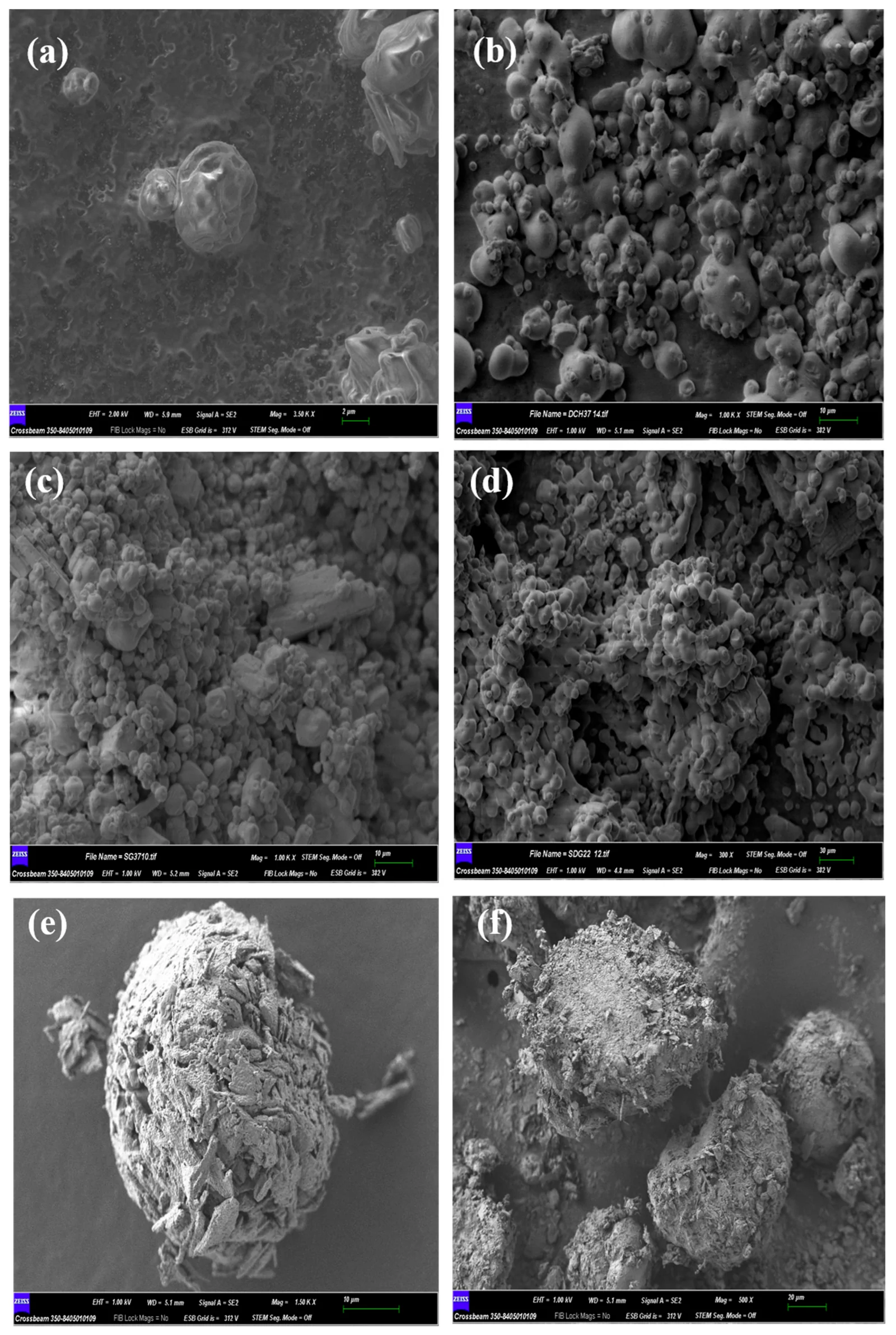
SEM micrographs of non-enriched (**a**,**b**): N-NV ((**a**), 3500×) and B-NV ((**b**), 1000×); pomegranate peel extract-enriched (**c**,**d**): N-PV ((**c**), 1000×), B-PV ((**d**), 300×); and commercial buffered references (**e**,**f**): B-C1 ((**e**), 1500×) and B-C2 ((**f**), 500×) at the end of storage time (month 6).

After 6 months of storage, the morphology of the spray-dried powders was generally preserved (Figure 2), since no particle collapse or structural deterioration was observed in the formulations. Thus, these results suggest that the drying process produced physically stable particles under the studied conditions, especially relevant in N-PV when the pomegranate peel extract was incorporated in the formulation.

The morphology of the buffered formulations was found to be slightly more regular than the unbuffered ones, consistent with other researchers who found a direct relationship between higher pH values (Table 2) and the production of microencapsulated particles with more defined surfaces [33]. On the contrary, commercial vinegars exhibited uneven surfaces with some edges and a fibrous appearance (Figure 1e,f). This irregular morphology could refer to a possible crystallization phenomenon during the drying process due to the migration of their components to the surface [34]. Although some limited particle aggregation was observed after 6 months in the buffered formulations (B-NV and B-PV), no significant cracking was exhibited after the storage period (Figure 2b,d). Thus, the preservation of particle morphology agrees with the physicochemical stability observed during storage, which supports the suitability of αCD as a carrier for maintaining the structural integrity of the spray-dried pomegranate peel extract-enriched powders.

### 2.2. Physicochemical Properties

#### 2.2.1. pH and Titratable Acidity

Table 2 shows the evolution of pH and titratable acidity (TA) of the evaluated powders during 6 months of storage at 22 °C. Non-buffered formulations (N-NV and N-PV) showed a acidic pH values at month 0 (3.4 ± 0.1 and 3.8 ± 0.1, respectively), consistent with their high content of free acetic acid and the absence of a buffering system. Since both formulations were prepared from the same liquid distilled vinegar, the significantly higher pH of the pomegranate peel extract-enriched powder (N-PV) compared with N-NV (*p* < 0.05) may be related to the incorporation of plant-derived solids during the UAE process prior to spray drying, which may have altered the composition of the feed solution. As expected, buffering with KHCO_3_ shifted the pH of the experimental buffered powders (B-NV and B-PV) towards near-neutral values (6.5 ± 0.1 and 6.3 ± 0.1, respectively) at month 0. Under these conditions, a substantial proportion of the free acetic acid was converted into potassium acetate, increasing pH while reducing the concentration of undissociated acetic acid in solution [35,36]. Comparable pH values were observed for the commercial buffered powders (5.9 ± 0.1 for B-C1 and 6.2 ± 0.1 for B-C2, month 0), consistent with the use of alkali-based buffering systems. The slight differences among buffered powders may reflect variations in formulation and overall ingredient composition. However, the specific contribution of individual components cannot be established for the commercial products, as their complete composition is not disclosed by the manufacturers.

In addition, the TA results were consistent with the pH measurements. N-NV showed the highest TA values throughout storage (8.2 ± 0.1% at month 0; 8.7 ± 0.3% at month 6), which is consistent with the preservation of the acidity of the original distilled vinegar during spray drying using αCD as an encapsulating carrier, in agreement with previous reports on spray-dried vinegar powders [15,23]. N-PV showed significantly lower TA values (5.8 ± 0.1% at month 0; *p* < 0.05). Rather than indicating acidity losses, this reduction may be attributable to the incorporation of the phenolic-rich pomegranate peel extract into the vinegar prior to atomization, which, although modifying the relative proportion of vinegar solids in the final powder, still preserved the characteristic acidic profile of the vinegar matrix.

The potassium-buffered experimental powders (B-NV and B-PV) presented the lowest TA values among all formulations (0.4 ± 0.1% and 0.3 ± 0.1% at month 0, respectively). These values should be interpreted considering the buffering process, as the conversion of free acetic acid into potassium acetate substantially reduced the measurable free acidity determined by titration. Consequently, the low TA values do not indicate the absence of acetate species in the powders but rather are consistent with the effectiveness of KHCO_3_ as a buffering agent [37]. From a technological perspective, buffered vinegar ingredients retain acetate species associated with their preservative functionality while reducing the proportion of free acid responsible for vinegar pungency, which may also influence the sensory characteristics of foods in which these new plant ingredients may be incorporated [16,17,19]. On the other hand, intermediate TA values were obtained for the commercial buffered powders (3.6 ± 0.1% for B-C1 and 3.2 ± 0.1% for B-C2, at month 0), indicating that a greater fraction of titratable acidity remained after buffering than in the experimental powders. Although direct comparisons should be interpreted cautiously because of differences in formulation and encapsulation systems, these results demonstrate that the use of potassium bicarbonate in the experimental formulations resulted in spray-dried powders with near-neutral pH and very low measurable acidity while reducing sodium incorporation, under the conditions evaluated in the present study [17,18].

Both pH and TA values remained stable throughout the 6-month storage period. N-NV maintained its high acidity (8.2–8.8%) with only minor pH variations, whereas B-NV preserved unchanged pH and TA values throughout storage (*p* > 0.05). Similarly, N-PV maintained stable acidity, while B-PV exhibited only slight increases in TA (0.3–0.6%) together with small pH variations that are unlikely to affect its physicochemical characteristics during storage. In contrast, B-C1 exhibited a gradual increase in pH accompanied by a slight decrease in TA, whereas B-C2 showed an initial reduction in TA followed by partial recovery during the remaining storage period. These findings indicate that neither the incorporation of the previously characterized pomegranate peel extract nor potassium buffering compromised the physicochemical stability of the experimental powders during storage. The stability observed after 6 months supports the use of spray-dried vinegar powders as a suitable matrix for incorporating phenolic-rich extracts recovered from plant byproducts, such as pomegranate peel.

#### 2.2.2. Moisture Content and Water Activity

Moisture content and water activity (a_w_) of the evaluated formulations are shown in Table 2. At the beginning of the storage time (month 0), the experimental spray-dried powders exhibited low moisture contents, ranging from 0.5–0.9%, below the 5% threshold generally considered suitable for the stability of spray-dried plant powders [28,38]. These values are consistent with those previously reported for spray-dried apple cider vinegar powder and nipa palm vinegar powders [15,23], indicating the efficiency of the spray drying process in producing low-moisture powders. In contrast, the commercial powder B-C1 presented a higher initial moisture content (1.8 ± 0.1%), which may be associated with differences in overall formulation and carrier composition, although the specific contribution of individual components cannot be established because the complete composition of the commercial product was not available.

Throughout storage, the moisture content of the experimental powders remained low, with no significant changes for most formulations (≤1.2% at month 6). Although B-PV reached 1.2 ± 0.1% after 6 months, this value was still lower than the critical level for spray-dried powders (<5%). The limited moisture uptake observed during storage is consistent with the limited increased in moisture content observed for the developed formulations under the evaluated conditions. The use of αCD as an encapsulating carrier may have contributed to this behaviour, as cyclodextrin-based matrices have been reported to contribute to reducing the hygroscopicity of spray-dried products by limiting moisture absorption during storage [25,27]. Conversely, both commercial powders showed a progressive increase in moisture content, reaching 3.0 ± 0.5% (B-C1) and 3.9 ± 0.6% (B-C2) after 6 months. The differences in overall formulation composition and particle surface characteristics (Figure 1) may have contributed to their greater hygroscopicity than that observed for the developed powders [34]. Nonetheless, the specific contribution of individual formulation components to this behaviour cannot be established, as the complete composition of the commercial products was not disclosed.

On the other hand, N-NV showed the highest initial a_w_ (0.47 ± 0.01), which was consistent with its comparatively higher moisture content (Table 2). Nevertheless, this value did not exceed the a_w_ value generally required for the growth of most pathogenic bacteria (a_w_ > 0.60), indicating favourable conditions for microbiological stability during storage [23]. Contrary to previous studies, despite the higher phenolic content in the pomegranate peel extract-enriched powder (N-PV) in comparison to N-NV, as discussed below, a considerably lower a_w_ was observed for the enriched formulation (0.26 ± 0.01) [24]. This formulation also exhibited a lower moisture content (0.5 ± 0.1% vs. 0.9 ± 0.1%), which together with its lower a_w_, may contribute to improved storage stability by helping preserve its antioxidant properties (TPC and TAC) under the tested storage conditions [39]. Nevertheless, a_w_ values were found to be similar to those previously reported for αCD spray-dried powders [27].

The potassium-buffered formulations (B-NV and B-PV) exhibited the lowest water activity values among all samples at the beginning of storage (0.18 ± 0.01 and 0.14 ± 0.01, respectively). During storage, the a_w_ decreased significantly during the first month and subsequently remained low, reaching 0.05 ± 0.01 for B-NV and 0.04 ± 0.01 for B-PV after 6 months (*p* < 0.05), despite only minor changes in moisture content. The more compact particle morphology observed for these formulations may be associated with the lower a_w_ values observed in the buffered powders, although the specific mechanisms underlying this relationship were not directly investigated in the present study [35,36]. On the contrary, the sodium-buffered commercial powders (B-C1 and B-C2) showed intermediate initial a_w_ values (0.26 ± 0.01 and 0.30 ± 0.01), followed by a significant gradual decrease to 0.21 ± 0.01 and 0.22 ± 0.01 after 6 months (*p* < 0.05). Simultaneously, both commercial powders exhibited a progressive increase in moisture content during storage (Table 2). These results may be consistent with the possibility that a proportion of the absorbed moisture became associated with the powder matrix rather than remaining as freely available water for microbial growth. Nonetheless, this proposed mechanism was not directly assessed in the present study. The more irregular and collapsed particles observed in SEM micrographs of the commercial powders have previously been associated with increased moisture adsorption in spray-dried powders owing to their larger effective surface area and greater exposure to environmental humidity [34]. Although direct mechanistic comparisons between the sodium-buffered commercial powders and the potassium-buffered experimental powders cannot be established because of their different compositions, the experimental buffered powders consistently maintained lower a_w_ values throughout storage while preserving similarly low moisture contents, thus supporting the suitability of the developed formulations as stable plant-derived ingredients under the evaluated storage conditions over the 6-month period, in comparison to the two commercial references.

#### 2.2.3. Color

**Figure 3:**
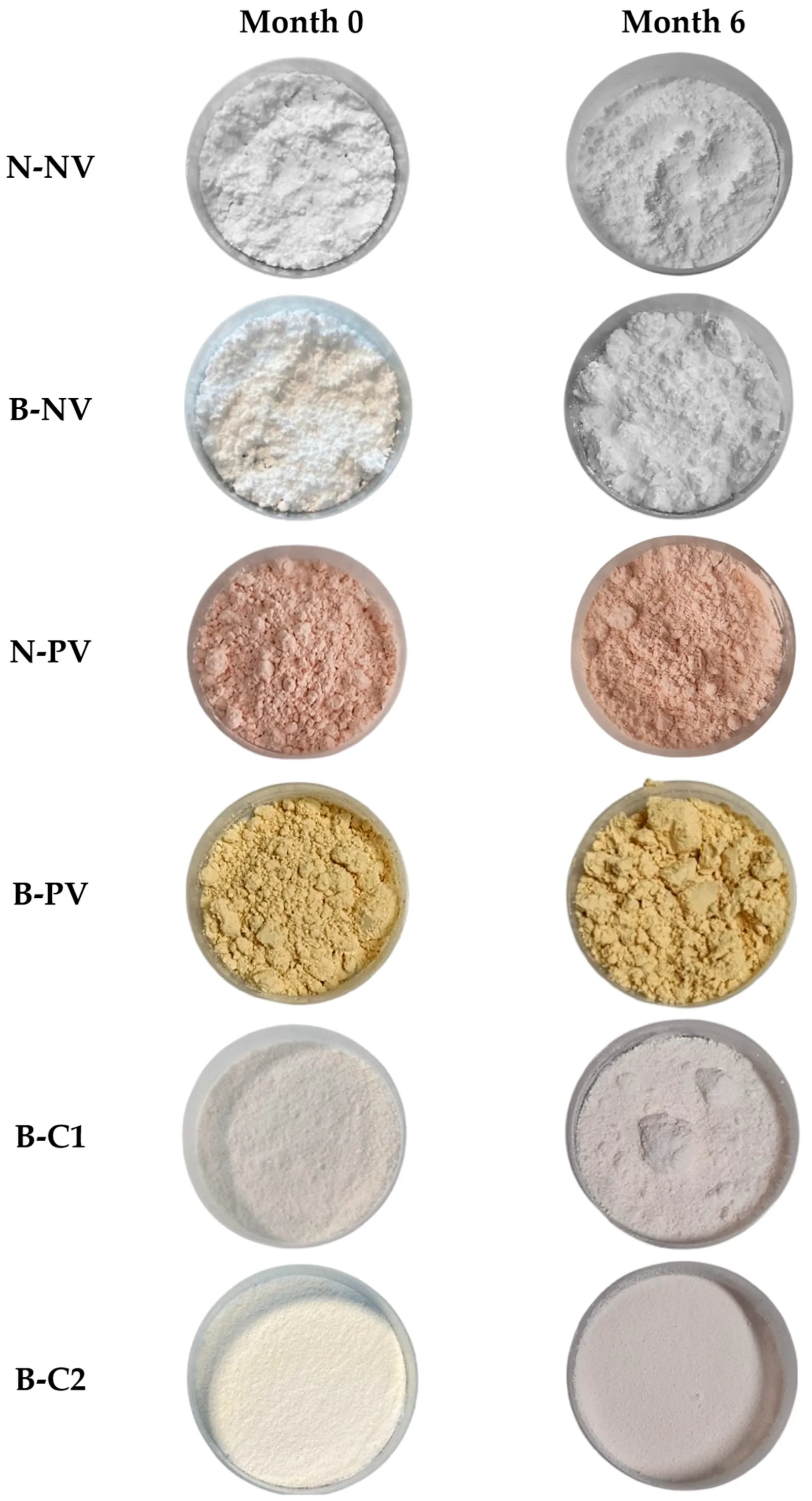
Appearance of non-enriched (N-NV and B-NV), pomegranate peel extract-enriched (N-PV and B-PV), and commercial buffered powders (B-C1 and B-C2) at the beginning of storage time (month 0) and after 6 months of storage at 22 °C.

Color values (L*, a*, b*) and total color differences (ΔE*) of the evaluated formulations throughout the 6-month storage time are presented in Table 3. At month 0, the non-enriched powders (N-NV and B-NV) showed higher luminosity (L* = 96.8–98.0) than pomegranate-enriched samples together with very low a* and b* values (a* = −0.1–0.0; b* = 0.3–1.3), which was consistent with the colourless nature of distilled vinegar and the white appearance of αCD used as encapsulating carrier during spray drying [23,40]. By contrast, the incorporation of pomegranate peel extract significantly influenced the initial color of the spray-dried powders (*p* < 0.05). N-PV showed lower luminosity but higher a* (+a*) and b* (+b*) values than N-NV, resulting in a moderately reddish and yellowish powder (Figure 3). These differences were associated with the incorporation of phenolic compounds and natural pigments from *Punica granatum* peel, which are responsible for the characteristic coloration of pomegranate-derived ingredients [22,41].

Buffering also influenced the color of the enriched powders, particularly in the formulations containing pomegranate peel-derived compounds. Compared with N-PV, B-PV exhibited lower L* (−L*) and a* (−a*) values, but significantly higher yellowness (+b*) (*p* < 0.05) (Figure 3). The increase in pH produced by potassium buffering influenced the color of the pomegranate-derived pigments, resulting in color shifts in the pomegranate-derived products under different pH conditions that have also been reported by other authors [24]. The commercial buffered powders also reflected the lack of color of the distilled vinegar used in their manufacture as well as the absence of coloured pigments derived from the pomegranate peel byproduct, since higher luminosity and moderate yellowness with a limited redness (−a*) were observed in B-C1 (Figure 3).

After 6 months of storage, the total color difference (ΔE*) remained below 3.5 for all formulations, indicating that the final color changes were limited [22]. During storage, the non-enriched powders (N-NV and B-NV) showed only minor variations and presented ΔE* values below 1.5 after 6 months, indicating barely perceptible color differences. Likewise, owing to the relatively stable color values of N-PV throughout storage, a low final color change (ΔE* = 1.7 ± 0.1) was observed. Therefore, the characteristic color imparted by the pomegranate peel extract in N-PV was largely preserved without compromising its stability during storage [22,42]. Previous studies have also reported improved color stability in fruit-derived powders of pomegranate encapsulated with different carbohydrate-based carrier mixtures [24,41].

However, greater color changes were observed for B-PV than for N-PV throughout storage. B-PV showed progressive variations in L*, a*, and b* values, reaching a maximum ΔE* value of 4.9 ± 0.2 at month 3, followed by a partial reduction to 3.3 ± 0.3 after 6 months. As pomegranate pigments are known to be sensitive to near-neutral conditions, the lower color stability of B-PV may be associated with the higher pH of this buffered formulation (Table 2) [24]. Under near-neutral pH conditions, pomegranate-derived pigments may undergo pH-dependent changes in their molecular forms, altering the relative abundance of coloured species and consequently affecting the a* and b* coordinates. This mechanism may explain, at least in part, the greater ΔE* observed for B-PV compared with N-PV, although the specific pigment transformations were not directly analysed in the present study. Although these changes were more pronounced than in the remaining experimental powders, the color values underwent minimal variation after month 3, thereby approaching stabilization during the rest of the storage period. The commercial buffered powders also exhibited notable color changes during storage, particularly B-C2 at month 6 (ΔE* = 3.4 ± 0.4), together with a gradual reduction in luminosity (−L*) and slight variations in the a* (−a*) and b* (+b*) values. B-C1 showed smaller color differences (ΔE* = 1.9 ± 0.4), indicating a comparatively more stable color profile throughout storage. Nevertheless, the greater color variation observed for B-C2 coincided with the higher moisture content measured after storage (Table 2), suggesting that moisture uptake may have influenced its color stability [24,42].

Despite the greater ΔE* observed in the developed buffered-enriched formulation (B-PV), significant preservation of the plant-derived pigments was observed under the evaluated storage conditions in N-PV powder, as limited color changes were observed for this particular formulation by the end of storage time [22].

### 2.3. Total Phenolic Content and Antioxidant Capacity

Total Phenolic Content (TPC) and Total Antioxidant Capacity (TAC) were evaluated throughout storage to estimate the evolution of antioxidant properties in the spray-dried formulations (Table 4). Formulation strongly influenced the antioxidant composition of spray-dried powders, especially when pomegranate peel extract was introduced. At month 0, pomegranate peel extract-enriched formulations (N-PV and B-PV) showed comparatively higher TPC (4.1–27.0 mg GAE/g_powder_) and TAC (38.0–287.8 µmol TE/g_powder_) than those obtained for the non-enriched (N-NV and B-NV) and commercial formulations (B-C1 and B-C2) (≤0.3 mg GAE/g_powder_ and ≤4.0 µmol TE/g_powder_, respectively). These results indicated that the pomegranate peel byproduct incorporated during the UAE process effectively increased the Folin–Ciocalteu-reactive fraction of the developed powders, which was accompanied by a higher antioxidant response, rather than as a result of the use of distilled vinegar as a liquid matrix. In fact, the phenolic composition of the corresponding liquid extracts was previously reported under the same extraction conditions [12]. In that study, punicalagin isomers were identified as the main phenolic compounds present in the liquid pomegranate peel extracts, together with ellagic acid derivatives, gallic acid and catechin, widely recognised as contributors to the antioxidant potential of pomegranate byproducts [10,12,20]. These individual phenolic compounds were not quantified in the spray-dried powders in the present study. Therefore, the TPC values obtained by the Folin–Ciocalteu assay should be interpreted as an estimate of the overall Folin–Ciocalteu-reactive fraction expressed as gallic acid equivalents, rather than as a direct quantification of punicalagin or other individual phenolic compounds. However, since the present results are expressed per gram of the final spray-dried powder, which also contains αCD as an encapsulating agent besides the other components of the respective formulations, they should not be directly compared with those reported for the liquid extracts [5]. Even so, the high antioxidant potential observed in N-PV indicates that the functional properties associated with the pomegranate peel extract were largely retained after spray drying. Comparable observations have been reported for other spray-dried ingredients obtained from pomegranate peel. Hadree et al. [43] found that increasing the proportion of pomegranate peel extract resulted in progressively higher TPC and antioxidant activity in the resulting spray-dried powders. Likewise, previous studies reported that appropriate spray-drying conditions preserved the antioxidant potential of pomegranate peel polyphenols after dehydration of liquid extracts [44]. Other researchers have reported similar TPC in spray-dried pomegranate peel ingredients ranging from 20–50 mg gallic acid/g [43], although formulation, carrier composition and processing conditions differed from those used here, these studies support spray drying as a suitable strategy to obtain stable antioxidant ingredients from pomegranate byproducts.

Although both enriched formulations were produced from the same pomegranate peel byproduct, B-PV showed a notably lower TPC (4.1 ± 0.2 mg GAE/g_powder_) and TAC (38.0 ± 1.1 µmol TE/g_powder_) than N-PV (27.0 ± 3.9 mg GAE/g_powder_ and 287.8 ± 5.6 µmol TE/g_powder_, respectively). At month 0, B-PV showed approximately 85% lower TPC and 87% lower TAC than N-PV. This difference is consistent with our previous study, in which the use of potassium-buffered vinegar during UAE resulted in lower recovery of selected pomegranate peel phenolic compounds than the corresponding non-buffered system [12]. In particular, the sum of the quantified phenolic compounds decreased from 76.2 ± 2.2 mg/g DW in the non-buffered-αCD vinegar system (V2-10) to 47.1 ± 1.7 mg/g DW in the potassium-buffered-αCD system (VT2-10), with a similar trend observed for punicalagin and other quantified phenolic compounds. Therefore, the lower TPC and TAC observed in B-PV may be mainly associated with differences established during the extraction stage rather than with the subsequent spray-drying process. The lower phenolic recovery in the buffered system may be related to the change in the extraction medium produced by KHCO_3_ neutralization. The resulting increase in pH and reduction in acidity may affect the ionization, solubility, and stability of individual phenolic compounds and consequently their recovery during UAE. However, the specific contribution of these factors was not directly investigated in the present study and therefore remains hypothetical. In addition, because TPC was expressed per gram of final spray-dried powder, differences in the contribution of buffering components to the final powder mass may also influence the magnitude of the difference between N-PV and B-PV.

During storage, TPC was substantially maintained for N-PV throughout the 6-month study (*p* > 0.05), while TAC showed only slight fluctuations over time. In particular, TAC in N-PV showed a significant decrease at month 3 compared with the initial value at month 0 (*p* < 0.05), although this variation was transitory, as the value at months 1 and 6 was not significantly different. This temporary decrease in TAC, in the absence of a significant change in TPC, may be related to changes in the availability of reducing compounds within the encapsulated matrix during storage, rather than to an actual loss of Folin–Ciocalteu-reactive compounds. B-PV followed a similar trend, showing stable TAC (*p* > 0.05) despite a modest increase in TPC after three months of storage. These results should therefore be interpreted cautiously, as they may reflect changes in the interactions between Folin–Ciocalteu-reactive compounds and the encapsulating matrix that may affect the measured antioxidant response without necessarily reflecting the formation of new phenolics. Regardless of the underlying mechanism, no substantial losses were observed in either TPC or TAC over the storage period, suggesting that the measured Folin–Ciocalteu-reactive and antioxidant capacities persisted under the evaluated conditions. Similar stability has been described for other spray-dried pomegranate powders encapsulated with carbohydrate-based carriers, where the protection provided by the encapsulating matrix contributed to the preservation of their antioxidant properties [22,24].

Pearson analysis revealed a very strong positive correlation between TPC and TAC (r = 0.994, *p* < 0.001), indicating that differences in radical scavenging capacity closely followed changes in the Folin–Ciocalteu-reactive fraction. This close relationship agrees with our previous findings [12] and with other studies on pomegranate-derived ingredients, supporting the contribution of phenolic compounds to the antioxidant activity of these formulations [5,22]. However, this correlation should not be interpreted as evidence of a contribution from any specific individual phenolic compound, since the Folin–Ciocalteu assay does not provide compound-specific quantification. These findings also support the use of TPC and TAC as suitable markers for monitoring the stability of measured antioxidant-related properties of the spray-dried formulations throughout storage. Enrichment with pomegranate peel extract enhanced the antioxidant properties of the developed powders (N-PV and B-PV), while the combination of UAE and spray drying with αCD proved suitable for obtaining stable functional ingredients containing pomegranate peel-derived polyphenols.

### 2.4. Antimicrobial Activity

Table 5 shows the inhibition zone diameters obtained for non-enriched (N-NV, B-NV), pomegranate-enriched (N-PV, B-PV), and commercial buffered vinegar powders (B-C1, B-C2) against *Listeria monocytogenes* STCC 4032 and *Salmonella enterica* STCC 443 at the beginning of storage time (month 0) and after 6 months at 22 °C. The non-enriched non-buffered formulation (N-NV) showed the largest inhibition zones against *L. monocytogenes* at both month 0 (29.6 ± 4.0 mm) and month 6 (28.4 ± 2.5 mm), with no significant differences (*p* > 0.05) from the positive control (31.4–31.5 mm). This behaviour agrees with the well-established antimicrobial activity of acetic acid, the main active compound in distilled vinegar. The undissociated form of acetic acid is able to diffuse through the bacterial membrane and cause intracellular acidification, disruption of metabolic processes and, ultimately, cell death [45,46]. The high acidity of the N-NV remained stable throughout storage (Table 2), which is consistent with the preservation of its antimicrobial activity [15]. Antimicrobial activity against microorganisms such as *Escherichia coli* or *Salmonella* Typhimurium has previously been reported for liquid vinegars evaluated by diffusion assays, where antimicrobial activity was positively associated with acetic acid concentration and low pH values [40,47].

As expected, buffering markedly reduced the antimicrobial activity of the developed formulations. Accordingly, B-NV produced inhibition zones of 9.5 ± 0.2 mm at month 0 and 8.7 ± 0.5 mm after 6 months of storage against *L. monocytogenes*, although above those generally considered indicative of relevant antimicrobial activity in agar diffusion assays (>7–10 mm) [47]. As a result of the buffering process, the proportion of undissociated acetic acid available to diffuse through the agar medium was decreased, thus reducing the antimicrobial potential of the buffered formulations [16,17]. Similarly, the commercial buffered powders exhibited reduced antimicrobial activity. B-C1 produced inhibition zones of 11.3 ± 1.2 mm and 11.3 ± 0.5 mm at month 0 and month 6, respectively, whereas limited inhibition was detected for B-C2 (7.3–7.5 mm). However, these results should be interpreted carefully, as agar diffusion assays mainly evaluate the diffusion capacity of antimicrobial compounds and may underestimate the effectiveness of buffered vinegar ingredients in food matrices, where antimicrobial activity develops under different conditions and acetate salts have previously demonstrated antimicrobial efficacy [16,19,48].

The incorporation of pomegranate peel extract influenced the antimicrobial activity of the buffered formulation. B-PV exhibited significantly larger inhibition zones against *L. monocytogenes* (24.9 ± 0.6 mm and 24.6 ± 0.6 mm at months 0 and 6, respectively) than B-NV and both commercial buffered powders (*p* < 0.05). The pomegranate peel extract used in the present study had previously been characterized as a rich source of phenolic compounds obtained by UAE, and its antimicrobial activity against *L. monocytogenes* and *S. enterica* was demonstrated before microencapsulation [12,22]. Although the mechanisms involved were not investigated in the present study, the results suggest that the incorporation of pomegranate peel extract may have partially compensated for the reduction in antimicrobial activity of the buffered formulation against *L. monocytogenes* [49]. This observation supports the potential of pomegranate peel as a source of bioactive compounds for the development of stable plant-derived ingredients.

Importantly, the same N-PV and B-PV spray-dried formulations evaluated in the present study were previously investigated in fresh-cut lettuce, providing complementary evidence of their performance in a real food matrix [50]. In that study, the formulations were applied both directly as powders and as washing treatments, and their effects were evaluated during refrigerated storage at 4 °C for up to 10 days. Vinegar treatments reduced microbial loads by approximately 2–4 log CFU g^−1^ after 10 days, while the washing treatments also contributed to maintaining the physicochemical and sensory quality of the lettuce. These findings demonstrate that the antimicrobial potential of these formulations can be observed beyond diffusion-based assays and under food-system conditions. However, the results obtained in that previous study should be considered complementary to the present findings, since the application conditions, food matrix, and experimental objectives differed from those of the present work.

Compared with the pomegranate peel-enriched liquid extract previously reported by our research group [12], the encapsulated powders showed lower inhibition zones against the same foodborne pathogens. Nonetheless, these results agree with previous studies indicating that microencapsulation by spray drying may reduce the apparent antimicrobial activity of phenolic-rich plant extracts in agar diffusion assays, as part of the bioactive compounds remain entrapped within the encapsulating matrix and thus limit their immediate diffusion through the agar medium [51]. Therefore, the lower inhibition zones observed for the spray-dried powders do not necessarily indicate a loss of antimicrobial potential but may partly reflect the slower release of encapsulated phenolic compounds during the assay. This consideration is particularly relevant when interpreting the antimicrobial activity of the present formulations, as the agar well-diffusion assay provides a measure of the compounds that are able to be released and diffuse through the agar under the specific experimental conditions, rather than a direct measure of their antimicrobial effectiveness in a food system.

By contrast, N-PV showed slightly smaller inhibition zones than N-NV at month 0 (26.5 ± 0.8 mm and 29.6 ± 4.0 mm, respectively), although no significant differences (*p* > 0.05) were observed between either formulation and the positive control (31.4 ± 1.9 mm). This slight reduction may be related to the lower relative proportion of vinegar solids in the enriched formulation following the incorporation of pomegranate peel extract before spray drying, rather than a loss of antimicrobial activity [11,20].

A different response was observed against *S. enterica*. Only the non-buffered formulations (N-NV and N-PV) produced measurable inhibition zones, whereas no inhibition was detected for any buffered formulation throughout storage. Furthermore, inhibition zones against *S. enterica* were substantially smaller than those observed against *L. monocytogenes*. This difference is consistent with the greater intrinsic resistance of Gram-negative bacteria to weak organic acids, generally attributed to the presence of a lipopolysaccharide-rich outer membrane that restricts the diffusion of antimicrobial compounds, together with acid tolerance mechanisms described for *Salmonella* spp. [52]. Likewise, although pomegranate-derived phenolic compounds have been reported to exhibit antimicrobial activity against Gram-negative bacteria, their effectiveness may be more limited under diffusion-based assays [49].

Together with the stability observed for acidity and antioxidant-related parameters, these results indicate that no marked loss of in vitro antimicrobial functionality occurred under the evaluated storage conditions. Thus, these results contribute to demonstrating that spray drying with αCD allowed the incorporation of pomegranate peel-derived bioactive compounds into a stable powdered ingredient while preserving their antimicrobial activity during storage. Nevertheless, further studies should investigate the antimicrobial effectiveness across different food matrices and storage conditions to better establish the relationship between in vitro activity and food-system performance.

### 2.5. Sensory Quality

#### 2.5.1. Sensory Evaluation of Reconstituted Spray-Dried Formulations

**Figure 4:**
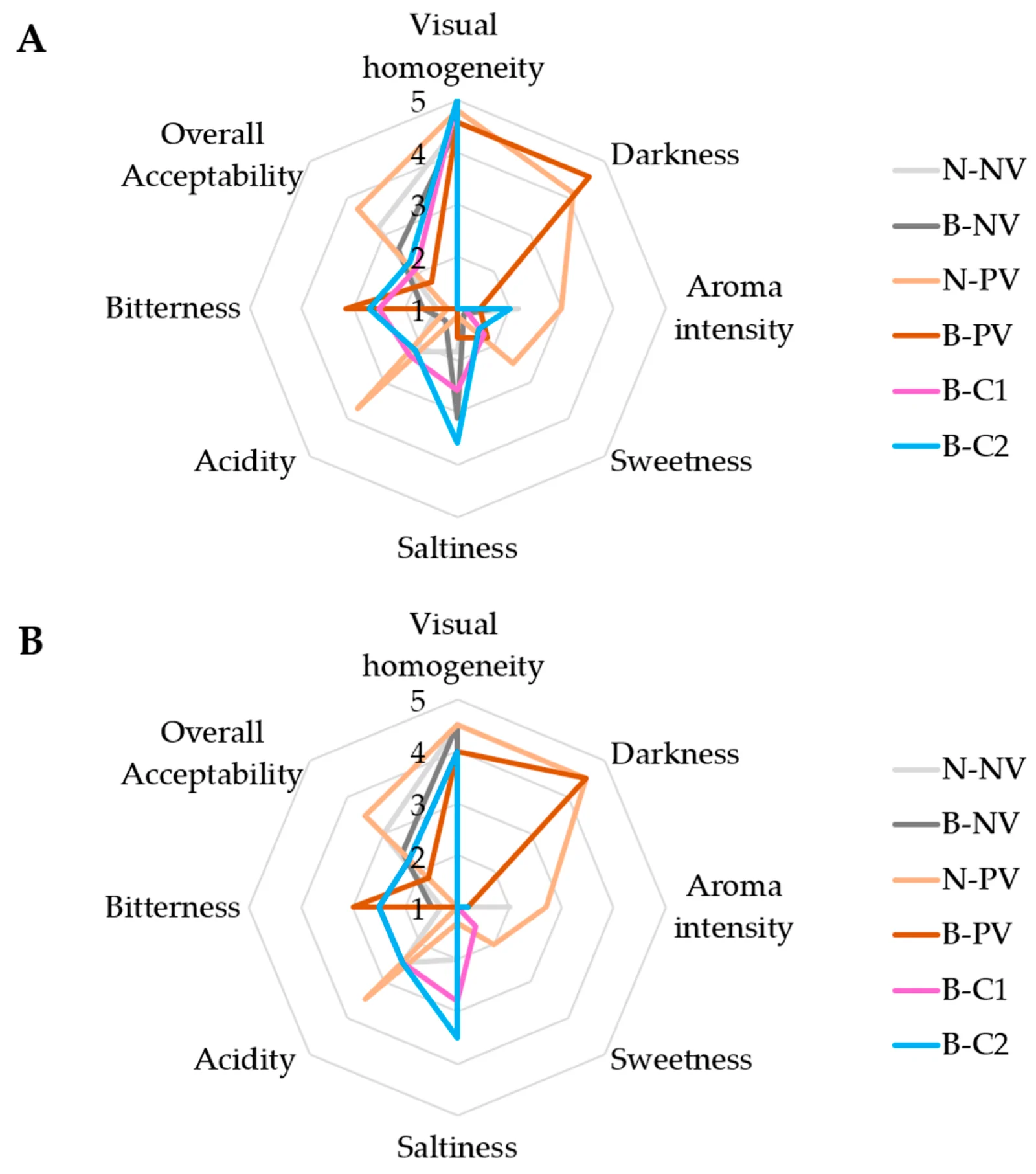
Sensory evaluation of the reconstituted spray-dried formulations immediately after production (month 0) (**A**) and after 6 months of storage at 22 °C (**B**): non-enriched (N-NV and B-NV), pomegranate peel extract-enriched (N-PV and B-PV), and commercial buffered powders (B-C1 and B-C2) (mean ± standard deviation, *n* = 14 assessors).

Figure 4 presents the results for the sensory evaluation of the reconstituted spray-dried formulations immediately after production (month 0) and after 6 months of storage at 22 °C. As the sensory evaluation was conducted with an untrained panel of 14 internal researchers, the results should be considered preliminary sensory acceptability screening rather than a consumer acceptance study. Therefore, the findings provide an initial indication of the sensory perception and acceptability of the formulations within the study panel and should not be extrapolated to the general consumer population. At both sampling times, all formulations were perceived as visually homogeneous and no visible sedimentation was observed after dispersion in water. In agreement with color measurements (Table 3), both enriched powders (N-PV and B-PV) were perceived as darker than the non-enriched formulations, as a result of the incorporation of the pomegranate peel extract. Nevertheless, this darker appearance did not negatively affect sensory perception and contributed positively to product acceptance, as N-PV obtained the highest overall acceptability score [53]. In addition, N-PV had a more intense and pronounced aroma than non-enriched and commercial formulations, which showed a more neutral aromatic profile instead, and was also perceived as more acidic than the rest of the samples (Figure 4A). This perception did not negatively affect sensory appreciation of the N-PV sample, as the natural sweet aromatic notes of pomegranate seemed to offset the perceived acidity and contributed to improving its acceptability [22]. In addition, the use of αCD as an encapsulating agent may have also contributed to the overall acceptability of the enriched non-buffered sample by masking phenolic astringency through inclusion complex formation [24,51]. These results indicate that the incorporation of pomegranate peel extract provided a more distinctive sensory profile while maintaining good preliminary sensory acceptability within the study panel.

Compared with N-PV, the buffered enriched formulation (B-PV) showed higher perceived saltiness and bitterness together with lower aroma intensity. A similar tendency towards increased saltiness was observed for the remaining buffered formulations, including the commercial references, reflecting the presence of buffering salts in these products. Notably, B-NV reached a saltiness perception comparable to that of B-C2 despite containing potassium rather than sodium as the buffering agent. Similar effects of spray-dried salt-containing formulations on salt perception have previously been reported [54,55]. Buffered formulations generally received slightly lower overall acceptability scores than non-buffered powders (N-NV and N-PV), which remained above the predefined acceptability threshold (score ≥ 3), indicating satisfactory sensory acceptance. Apart from that, non-enriched and commercial formulations exhibited a more neutral aromatic profile compared to N-PV. This characteristic, coupled with low perceived acidity, may have contributed to their lower sensory acceptance.

Visual homogeneity was maintained in every formulation, just as the differences observed between formulations were generally preserved throughout storage (Figure 4B). N-PV continued to receive the highest overall acceptability scores, whereas the buffered formulations maintained higher perceived saltiness than the non-buffered samples. Individual sensory attributes underwent minimal changes, without evident deterioration in aroma perception, flavor or overall acceptability. These observations were consistent with the limited physicochemical changes observed during storage, particularly regarding color and titratable acidity. Furthermore, the studied storage period of 6 months at 22 °C did not substantially modify the sensory characteristics of the formulations, supporting the preservation of the sensory attributes associated with the pomegranate peel extract under these conditions.

#### 2.5.2. Sensory Evaluation of Croquettes Containing the Spray-Dried Formulations

**Figure 5:**
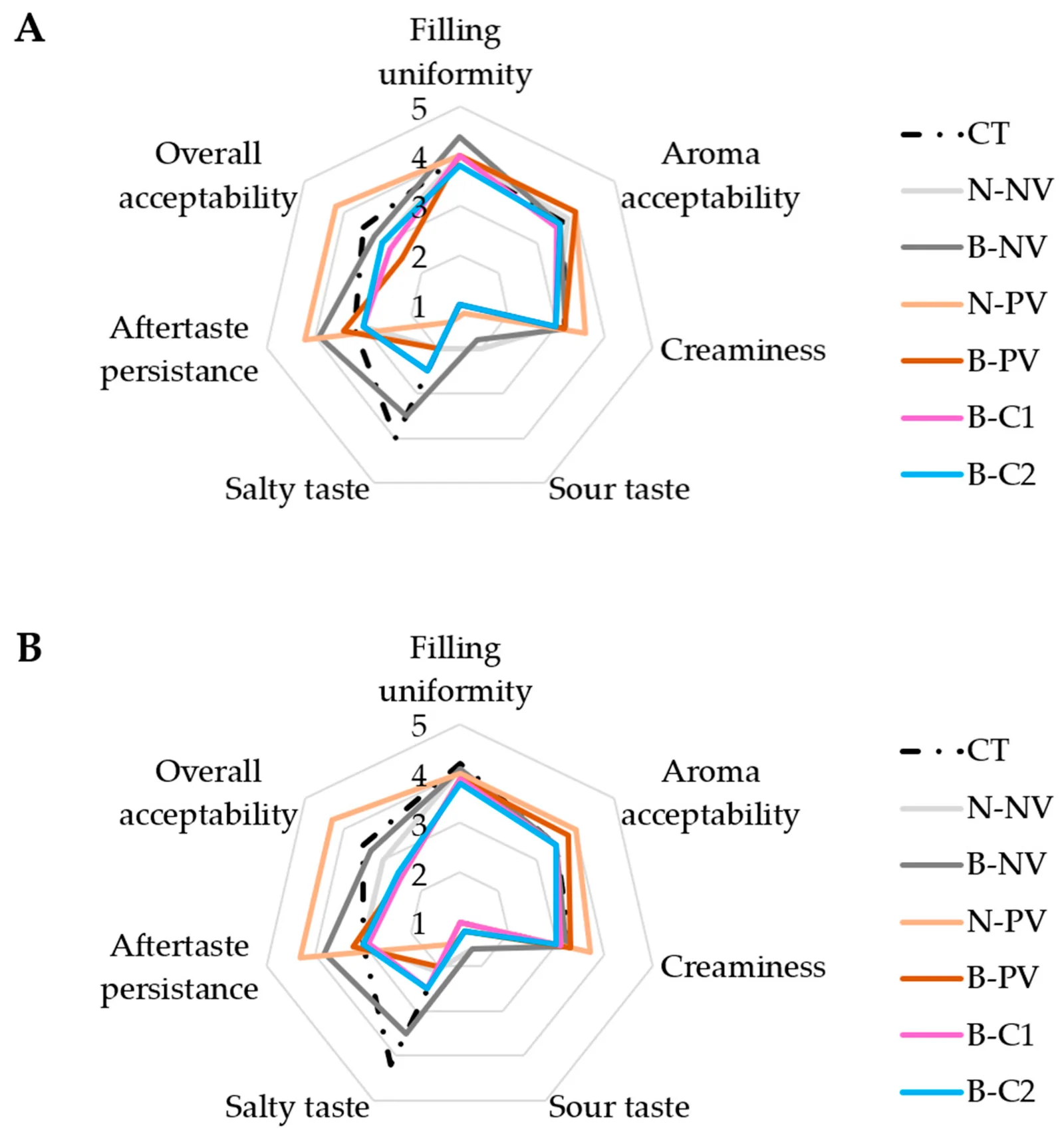
Sensory evaluation of croquettes prepared with the developed spray-dried formulations immediately after production (month 0) (**A**) and after 6 months of storage at 22 °C (**B**): non-enriched (N-NV and B-NV), pomegranate peel extract-enriched (N-PV and B-PV), and commercial buffered powders (B-C1 and B-C2). Control formulation prepared with NaCl that did not incorporate spray-dried powder was included (CT) (mean ± standard deviation, *n* = 14 assessors).

Figure 5 shows the sensory evaluation of croquettes prepared with the developed spray-dried formulations (non-enriched and pomegranate peel extract-enriched), in comparison to a control formulation without spray-dried powder (CT) and the two commercial buffered powders, both at month 0 and after 6 months of storage. As for the reconstituted formulations, this assessment represents a preliminary sensory acceptability screening rather than a consumer acceptance test, given the use of the same untrained internal panel. In general, the incorporation of the spray-dried powders mainly influenced flavor perception and overall acceptability, whereas filling uniformity and aroma acceptability were similar among formulations throughout storage. Croquettes formulated with the non-buffered enriched powder (N-PV) received the highest overall acceptability scores at both month 0 and month 6, together with high aroma acceptability, creaminess and aftertaste persistence. The enriched formulations also exhibited lower perceived sourness than the corresponding non-enriched powders, suggesting that the incorporation of pomegranate peel extract positively contributed to the sensory profile of the croquette product, although flavor perception was probably influenced by interactions between the powder composition and the food matrix [9,22]. Buffered samples also showed slightly lower sourness than the non-buffered ones, consistent with the role of buffering salts in moderating the perceived acidity of vinegar [16,17] (Figure 5A).

As expected, CT croquettes obtained the highest salty taste score. Nevertheless, it must be noted that only CT croquettes incorporated NaCl directly as the only seasoning agent in the filling dough, while this condiment was replaced by the spray-dried powder in the rest of the samples. However, it is worth noting that among the developed spray-dried formulations, buffered samples (B-NV and B-PV) were perceived as saltier than their corresponding non-buffered samples (B-NV > N-NV and B-PV > N-PV), in agreement with the contribution of potassium salts to saltiness previously reported for buffered vinegar ingredients and potassium chloride-based salt replacers [17,18]. Additionally, B-NV maintained overall acceptability values comparable to those of the control and the commercial references, suggesting that the incorporation of potassium-buffered powder did not negatively affect the sensory quality of the croquettes under the evaluated conditions. Similar improvements in salt perception have previously been associated with other spray-dried salt-containing formulations [54,55].

On the other hand, N-PV showed the highest creaminess scores at both storage times, whereas buffered formulations exhibited slightly lower values, particularly the B-PV formulation. Aroma acceptability remained notably stable among formulations and throughout storage, indicating that neither pomegranate peel enrichment nor buffering adversely affected the aromatic perception of the final product. N-PV also received the highest aftertaste persistence scores, followed by B-NV, whereas the commercial references showed comparatively lower values. The more persistent aftertaste perceived for N-PV may be associated with the incorporation of pomegranate peel-derived compounds into the spray-dried formulation, since αCD has been reported to form inclusion complexes that can modulate the release and perception of bioactive compounds [11,27]. N-PV remained the most appreciated formulation by the end of the storage time, whereas the commercial buffered powders showed lower overall acceptability (Figure 5B).

Overall, the incorporation of the pomegranate peel extract into the spray-dried formulation resulted in a sufficient sensory acceptance when applied to a model food product, such as croquettes. These results contribute to demonstrating the combined use of αCD and spray drying as a technological strategy to mask undesirable flavors while simultaneously preserving and enhancing pleasant aromatic notes in the evaluated croquettes [22,24]. The non-buffered enriched formulation (N-PV) achieved the highest preliminary sensory acceptability while maintaining its sensory quality after 6 months of powder storage, thus supporting the potential of pomegranate peel byproducts as ingredients for the development of stable plant-derived food formulations [16,48]. Nevertheless, the sensory findings should be interpreted with caution given the small size and untrained nature of the internal panel. Future studies should include a larger and more representative consumer panel to assess consumer acceptance, complemented, where appropriate, by trained sensory analysis to provide a more detailed characterization of the sensory attributes of the developed formulations.

## 3. Materials and Methods

### 3.1. Plant Material and Sample Description

Dehydrated pomegranate (*Punica granatum* L.) peel powder obtained from the Spanish cultivar Mollar de Elche was provided by Agrosingularity S.L (Murcia, Spain). The same batch of plant material previously used for the preparation and characterization of the pomegranate peel extract reported by Martínez-Sánchez et al. [12] was employed in the present study. Liquid distilled vinegar with high acetic acid content (≥20%) was supplied by JR Sabater S.A. (Murcia, Spain). α-cyclodextrin (αCD) (Wacker Chemie AG; Burghausen, Germany) was used as extraction co-solvent and encapsulating agent. Two commercial distilled buffered vinegar powders were included as reference samples: Verdad^®^ Powder N6 (Corbion; Amsterdam, The Netherlands) and BactoCEASE™ NV DRY (EU) (Kemin Food Technologies; Veronella, Italy).

### 3.2. Preparation of Spray-Dried Pomegranate Peel Extract-Enriched Vinegar Powders

**Figure 6:**
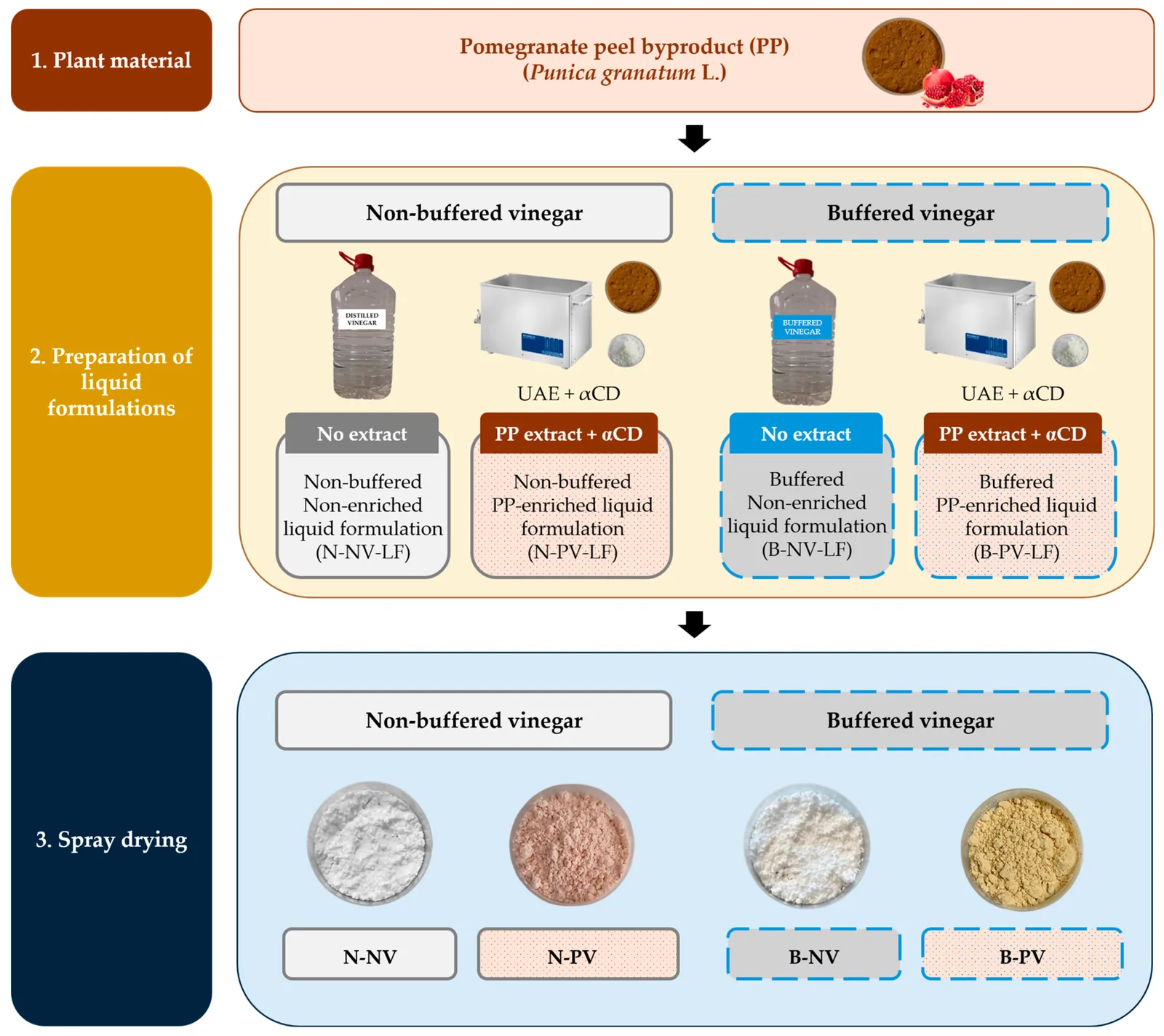
Experimental workflow for the preparation and spray drying of non-enriched (N-NV and B-NV) and pomegranate peel extract-enriched (N-PV and B-PV) powder formulations. PP = pomegranate peel byproduct; UAE = ultrasound-assisted extraction; αCD = α-cyclodextrin; LF = liquid formulation; N-NV = non-buffered non-enriched vinegar powder; N-PV = non-buffered pomegranate peel extract-enriched vinegar powder; B-NV = buffered non-enriched vinegar powder; B-PV = buffered pomegranate peel extract-enriched vinegar powder.

Liquid distilled vinegar was employed to prepare the non-enriched formulations (non-buffered and buffered) (Figure 6). Buffering of liquid distilled vinegar was carried out by the gradual addition of potassium salts (KHCO_3_) under continuous stirring until pH 6.2 was reached. During neutralization, the temperature was maintained between 35–54 °C to minimize acetic acid losses, as previously described [35,36].

For the enriched formulations, pomegranate peel extracts were obtained from the pomegranate peel byproduct described in Section 3.1 by ultrasound-assisted extraction (UAE) using an ultrasound device Bandelin Sonorex Digiplus DL 514 BH (Berlin, Germany) with distilled vinegar (non-buffered or buffered) and 10% αCD (*w*:*v*), following the methodology previously reported by our research group [12] (Figure 6). That study evaluated the extraction conditions and characterized the resulting liquid extracts in terms of phenolic profile by HPLC-QTOF-MS, antioxidant capacity, and antimicrobial activity. The same extraction procedure and formulations were used in the present work, where the liquid extracts were subsequently spray-dried and their storage stability was evaluated.

Prior to spray drying, the pomegranate-enriched extracts were filtered through a 0.106 mm stainless steel sieve (∅200 mm, according to ISO 3310-1:2000 [56]; mesh material AISI 316 and frame material AISI 304; Filtra^®^ Filtra Vibración S.L.; Barcelona, Spain), to remove coarse particles and prevent nozzle obstruction. Non-enriched formulations were mixed directly with 10% αCD (*w*:*v*) before atomization. Spray drying was performed by Bio-iPack (Bioencapsulation and iPackaging S.L., Fuente Álamo Technology Park; Fuente Álamo, Spain) using a pilot-scale spray dryer (manufactured by the company Mecánicas Bolea S.A.; Cartagena, Spain). The αCD concentration was selected according to preliminary optimization studies. The spray drying process was performed under the following conditions: 160 ± 2 °C inlet temperature, 85 ± 2 °C outlet temperature, 30 mL/min liquid feed rate, 90 m^3^/h spraying hot air flow rate, and 4 bar atomization pressure, respectively. After that, four experimental powders were obtained (Table 6): non-buffered non-enriched (N-NV), buffered non-enriched (B-NV), non-buffered pomegranate-enriched (N-PV), and buffered pomegranate-enriched (B-PV). Two commercial buffered non-enriched vinegar powders were included as reference samples.

### 3.3. Storage Conditions and Shelf-Life Study

Non-buffered and buffered vinegar powders (non-enriched, pomegranate peel extract-enriched, and commercial formulations) were hermetically sealed in 30 mL amber glass screw-cap bottles (P28), with 8 g of powder per bottle. All bottles for each formulation were filled from the same spray-drying batch obtained in a single spray-drying run. Three independently sealed bottles were evaluated at each storage time. For each replicate, the required amount of powder was withdrawn from one individual bottle and used for the corresponding analyses. Thus, the three replicates represent independent storage units from the same spray-drying batch. Samples were stored at 22 ± 1 °C for 6 months under standard laboratory conditions (Incucell ID 222; Munich, Germany). Morphological characterization (particle size distribution and scanning electron microscopy) and sensory evaluation were performed on freshly prepared powders (month 0) and after 6 months of storage. Physicochemical properties (pH, titratable acidity, moisture content, water activity and color) together with antioxidant parameters (total phenolic content and DPPH radical scavenging activity) were evaluated at months 0, 1, 3 and 6. Antimicrobial activity against *Listeria monocytogenes* STCC 4032 and *Salmonella enterica* STCC 443 was determined at month 0 and after 6 months of storage.

### 3.4. Morphological Characterization Methods

#### 3.4.1. Particle Size Distribution Analysis

The particle size distribution of the non-enriched and pomegranate-enriched powdered vinegar was measured by wet method using a laser diffraction particle size analyzer (Mastersizer 2000, Malvern Instruments; Malvern, UK) coupled to a Hydro 2000SM accessory unit. Particle size distributions were obtained (0.06–2000 µm) after dispersion of 0.2 g of sample in ethanol. For each measurement, D_10_, D_50_ and D_90_, and Span parameters were obtained.

#### 3.4.2. Scanning Electron Microscopy (SEM)

Morphology of powdered formulations was studied by scanning electron microscopy (SEM). A scanning electron microscope (Hitachi S-3500N, Hitachi; Tokyo, Japan) with a tungsten filament electron source was used to record black and white micrographs, with automatic polarization and electronic alignment of the electron gun with an accelerating voltage of 0.5–30 kV and a magnification range of 18–300,000×.

### 3.5. Physicochemical Characterization

#### 3.5.1. Determination of pH and Titratable Acidity

For the determination of pH and titratable acidity, 1 g of powder was dispersed in 10 mL of distilled water. The pH of the resulting suspension was measured using a digital pH meter (GLP 21, Crison; Barcelona, Spain). Titratable acidity was determined using an automated titrator (916 Ti-Touch, Metrohm; Herisau, Switzerland) with 0.1 M NaOH until an endpoint of pH 8.1. Titratable acidity was expressed as grams of acetic acid per 100 g of spray-dried powder (% *w*/*w*).

#### 3.5.2. Moisture Content

The moisture content of the powdered formulations was determined using the AOAC gravimetric method [57], with some adaptations. Briefly, 50 mg of each sample were heat-sealed into food-grade plastic vacuum bags (5.7 × 5.4 cm) and pierced twice with a sterile needle prior to overnight drying at 70 ± 1 °C inside a vacuum oven (Binder VD 23; Tuttlingen, Germany) until constant weight. The moisture content (%) was calculated as indicated in Equation (1):

(1) $$Moisture\;content\;\left(\%\right)=\;\frac{W_{f}-W_{d}}{W_{f}}\times 100$$

where *W_f_* is the initial weight of the sample (before drying) and *W_d_* is the weight of the sample after drying.

#### 3.5.3. Water Activity

A water activity analyzer (Labmaster-aw “basic” Novasina AG; Lachen, Switzerland) was used for measurement of the water activity of the powdered formulations [38]. Readings were taken at room temperature (22 ± 1 °C), recording the average of three replicates per sample.

#### 3.5.4. Color Measurement

The color determination of the powdered formulations was evaluated using a colorimeter (CR-400 Chroma Meter, Konica Minolta; Tokyo, Japan) at CIE illuminant C and 2° observer with an 8 mm diameter measuring aperture, previously calibrated (Y = 94.3; x = 0.3142; y = 0.3211). Color values (L*, a*, and b*) were reported as the mean value of three readings per replicate. Color changes during storage were calculated as the Total Color Differences (ΔE*) using Equation (2) [42]. The values ΔE* were calculated individually for each replicate using the corresponding L*, a*, and b* values at each storage time point relative to the initial measurement (month 0). Subsequently, the mean and standard deviation of the individual ΔE* values were calculated and reported.

(2) $$\Delta E*=\sqrt{({\Delta L*)}^{2}+{\left(\Delta a*\right)}^{2}+{\left(\Delta b*\right)}^{2}}$$

### 3.6. Determination of Total Phenolic Content

Total Phenolic Content (TPC) was determined using the Folin–Ciocalteu method described by Singleton and Rossi [58], with minor modifications. The Folin–Ciocalteu assay provides an estimate of the reducing capacity of the sample under the assay conditions and is not specific to individual phenolic compounds. TPC was used to monitor the stability of the overall Folin–Ciocalteu-reactive fraction of the spray-dried formulations during storage. The phenolic profile of the liquid pomegranate peel extracts used for spray drying had been previously characterized by HPLC-QTOF-MS under the same extraction conditions [12]. Therefore, the present study focused on monitoring the preservation of the overall Folin–Ciocalteu-reactive fraction of the encapsulated formulations during storage rather than on the identification or quantification of individual phenolic compounds in the spray-dried powders.

Spray-dried powder (100 mg) was homogenized with an aqueous mixture of methanol-Milli-Q^®^ ultrapure type 1 water (1:1, *v*:*v*) at 120 rpm/1 h (Stuart; Staffordshire, UK) in cold (4 °C) and dark conditions. Samples were centrifuged (14,000× *g*/5 min/4 °C) (Sorvall Legend Micro 17R, Thermo Scientific; Waltham, MA, USA), and TPC analysis was performed employing turbidity-free supernatants. Aliquots of the resulting extracts were inserted into a 96-well microtiter plate (Greiner Bio-One; Frickenhausen, Germany) and incubated with a 0.5 N working solution of Folin–Ciocalteu reagent, prepared by diluting the commercial 2 N reagent (Sigma-Aldrich; St. Louis, MO, USA) for 3 min in dark conditions. Samples were then added with a solution of 0.4% NaOH and 2% Na_2_CO_3_ (PanReac AppliChem ITW Reagents; Barcelona, Spain) and incubated at room temperature and dark for 2 h. Absorbance was read at 750 nm using a microplate reader (Infinite M Plex 200 Pro, Tecan Trading AG; Männedorf, Switzerland). TPC was expressed as mg gallic acid equivalents (GAE)/g_powder_ using a gallic acid calibration curve (0–1 mM).

### 3.7. Determination of Antioxidant Activity

Antioxidant activity was determined using the DPPH free radical scavenging assay according to Brand-Williams et al. [59], with adaptations. The same methanolic extracts obtained for TPC determination were used for DPPH analysis to monitor the preservation of the antioxidant capacity of the spray-dried formulations during storage.

Aliquots of the extracts were transferred into a 96-well microtiter plate (Greiner Bio-One; Frickenhausen, Germany) and mixed with a DPPH solution (2,2-diphenyl-1-picrylhydrazyl, 95% purity; Sigma-Aldrich; St. Louis, MO, USA) previously adjusted to an absorbance of 1.10 ± 0.02 at 515 nm. After incubation for 25 min at room temperature in the dark, absorbance was measured at 515 nm using a microplate reader (Infinite M Plex 200 Pro, Tecan Trading AG; Männedorf, Switzerland). Antioxidant activity was expressed as µmol Trolox equivalents (TE)/g_powder_ using a Trolox ((±)-6-Hydroxy-2,5,7,8-tetramethyl-chromane-2-carboxylic acid, 97% purity; Sigma-Aldrich; St. Louis, MO, USA) calibration curve (0–1 mM).

### 3.8. Determination of Antimicrobial Activity

Agar well-diffusion method [60], with some adaptations, was selected for the evaluation of the antimicrobial activity of the powdered formulations. *Listeria monocytogenes* STCC 4032 and *Salmonella enterica* STCC 443 lyophilized pathogenic strains were obtained from the Spanish Type Culture Collection (STCC). Previously, both bacteria were activated according to STCC specifications, making two passages in TSB (Tryptic Soy Broth, Scharlau Chemie; Barcelona, Spain) (37 °C/20 h per passage) until 10^8^ CFU/mL. Three wells (7.0 ± 0.1 mm diameter) were done on PCA (Plate Count Agar, Scharlau Chemie; Barcelona, Spain) solid agar plates (4–5 mm thickness) after inoculation of 0.1 mL of 10^6^ CFU/mL bacterium. After that, 50 µL of sample or control (positive or negative) was added to each corresponding well. Reconstituted solutions were prepared by diluting spray-dried samples in sterilized distilled water (1:1, *w*:*v*). As positive control was used a broad-spectrum antibiotic, oxytetracycline dihydrate (Sigma-Aldrich; St. Louis, MO, USA) (100 mg/L, *w*:*v*); as negative control, sterilized distilled water was employed. The inhibition zone diameter (mm) was measured with a digital caliper after 24 h of incubation at 37 °C. All determinations were performed in triplicate.

### 3.9. Sensory Evaluation

#### 3.9.1. Sensory Evaluation Procedure for Reconstituted Spray-Dried Formulations

Sensory analysis was conducted at the Institute of Plant Biotechnology (IBV; Cartagena, Spain), in a sensory evaluation room equipped with eight individual booths. An untrained panel consisting of 14 researchers participated in the study (9 female and 5 male). The age distribution of the panelists was 20–30 years (*n* = 9), 31–40 years (*n* = 3) and 41–50 years (*n* = 2). Sensory evaluation was performed on the reconstituted powder formulations (non-enriched, pomegranate peel extract-enriched, and commercial formulations) according to the organoleptic methodology described by Kang et al. [61]. The formulations were reconstituted at 5% (*w*:*v*) in bottled mineral water and served at room temperature in transparent tubes identified with random three-digit codes. The reconstituted formulations were evaluated to assess their intrinsic sensory characteristics independently of a food matrix. Each panelist evaluated the six formulations in a randomized order and received written instructions before the evaluation.

Visual homogeneity, darkness, aroma intensity, sweetness, saltiness, acidity, and bitterness were evaluated using a five-point intensity scale, where 1 and 5 represented the lowest and highest perceived intensity, respectively. Overall sensory acceptability was assessed using a five-point hedonic scale, where 1 corresponded to “not acceptable” and 5 to “excellent”. A score of 3 was considered as a descriptive acceptability threshold for this preliminary sensory screening, with lower values indicating lower acceptability within the panel. Bottled mineral water and unsalted breadsticks were provided for rinsing the mouth and preventing sensory fatigue between samples. No compensation was provided for participation in the sensory evaluation.

#### 3.9.2. Sensory Evaluation of Croquettes Containing Pomegranate Peel Extract-Enriched Powder Formulations

To assess the incorporation of the developed spray-dried formulations into a food model matrix, a preliminary sensory acceptability screening was carried out using béchamel croquettes as a model product.

Croquettes were prepared following the procedure described in [62], with minor modifications. The basic ingredients were purchased from a local supermarket (Cartagena, Spain). Xanthan gum was supplied by Doscadesa S.L. (Murcia, Spain) and wheat breadcrumbs were obtained from Frumen Desarrollos Alimentarios S.A (Guadalajara, Spain). Seven croquette formulations were prepared: a control formulation (CT, without powder), non-enriched formulations (N-NV and B-NV), pomegranate peel extract-enriched formulations (N-PV and B-PV), and two commercial reference formulations (B-C1 and B-C2). The powder formulations were incorporated into the croquette filling, whereas the control formulation contained NaCl instead of powder. The dough was cooled down at room temperature and refrigerated (4 °C, 45 min) prior to individually molding it by hand into cylindrical croquettes inside a laminar flow cabinet (ISO 5 [63]; equivalent to 100 FED STD 209E class) located in a cleanroom (ISO 7; equivalent to 10,000 FED STD 209E class) to ensure food safety. Croquettes were coated with industrial albumen and wheat breadcrumbs, frozen at −20 °C for 24 h, and baked in a household hot-air oven (model 3HB4841X0, BSH; Zaragoza, Spain) at 180 °C for 10–15 min before serving.

Sensory evaluation was conducted in the individual booths of the IBV (Cartagena, Spain) using the same untrained panel described above. Samples were served on disposable plastic plates identified with random codes. Appearance (filling uniformity) and aroma acceptability were evaluated before tasting, whereas creaminess, sourness, saltiness, aftertaste persistence, and overall acceptability were assessed during and after consumption using a five-point hedonic scale (1 = “extremely bad”; 3 = “acceptable”; and 5 = “excellent”). Written instructions were provided before the evaluation. Bottled mineral water and unsalted breadsticks were supplied for palate cleansing between samples.

### 3.10. Statistical Analysis

Statistical analysis was performed using R software (v.4.2.3) through RStudio software (v.2023.03.0 Posit Software; Boston, MA, USA). Data were analyzed at a 95% confidence level (*p* < 0.05) using two-way analysis of variance (ANOVA), followed by Tukey’s HSD test. When necessary, data were transformed using the natural logarithm to meet the assumptions of normality and homogeneity of variances. Pearson correlation analysis was performed to evaluate the relationship between total phenolic content (TPC) and antioxidant capacity (TAC). For the analytical determinations performed on the stored powders, results are presented as the mean value ± standard deviation of three independent storage replicates per sample and sampling time. The three replicates corresponded to three separately sealed bottles filled from the same spray-drying batch and were not independent spray-drying process replicates. Sensory results are presented as mean ± standard deviation based on the scores provided by the 14 assessors. Sensory comparisons were considered exploratory and were used to compare the observed panel scores among formulations and evaluation conditions.

## 4. Conclusions

Pomegranate peel is a major byproduct generated of the fruit-processing industry and represents an abundant source of phenolic compounds with well-documented antioxidant potential. In this study, spray-dried vinegar powders enriched with pomegranate peel extract previously obtained by ultrasound-assisted extraction (UAE) using vinegar and α-cyclodextrin (αCD) were evaluated during 6 months of storage at 22 °C. The incorporation of pomegranate peel extract into non-buffered and buffered vinegar powders markedly enhanced their antioxidant properties compared with the non-enriched and commercial formulations. Additionally, TPC and TAC were notably preserved throughout the 6-month period, while the powders also exhibited suitable physicochemical and morphological characteristics under the evaluated storage conditions. Sensory evaluation also showed preliminary sensory acceptability of the developed formulations, both after reconstitution and after their incorporation into a model food matrix. The powders exhibited in vitro antimicrobial activity under the well-diffusion assay conditions; however, their preservative efficacy in real food systems was not evaluated in the present study. These findings indicate that the combination of UAE and spray drying using vinegar and αCD represents a promising strategy for the valorization of pomegranate peel byproducts and the development of stable powdered plant-derived ingredients with preserved antioxidant properties and in vitro antimicrobial activity associated with the vinegar-based formulations under the studied conditions.

## Figures and Tables

**Table 1:** Particle size distribution (D_10_, D_50_, D_90_ and Span) of non-enriched (N-NV and B-NV), pomegranate peel extract-enriched (N-PV and B-PV), and commercial buffered powders (B-C1 and B-C2) during standard storage at 22 °C for up to 6 months (mean (*n* = 3) ± standard deviation).

Time (Month)	Treatment	D_10_ (µm)	D_50_ (µm)	D_90_ (µm)	Span
0	N-NV	6.9 ± 0.1 ^Aa^	59.8 ± 0.1 ^Aa^	131.9 ± 0.1 ^Aa^	2.1 ± 0.1 ^Aa^
	B-NV	7.5 ± 0.1 ^Ba^	29.2 ± 0.1 ^Ba^	379.9 ± 0.1 ^Ba^	12.5 ± 0.3 ^Ba^
	N-PV	4.2 ± 0.1 ^Ca^	26.1 ± 0.1 ^Ca^	391.5 ± 0.1 ^Ca^	14.8 ± 0.1 ^Ca^
	B-PV	8.4 ± 0.1 ^Da^	31.8 ± 0.1 ^Da^	338.0 ± 0.1 ^Da^	10.4 ± 0.1 ^Da^
	B-C1	156.8± 0.1 ^Ea^	480.5 ± 0.1 ^Ea^	853.7 ± 0.2 ^Ea^	1.4 ± 0.1 ^Ea^
	B-C2	54.8 ± 0.1 ^Fa^	109.6 ± 0.1 ^Fa^	191.7 ± 0.2 ^Fa^	1.3 ± 0.1 ^Ea^
6	N-NV	5.8 ± 0.2 ^Ab^	30.7 ± 0.6 ^Ab^	214.8 ± 0.1 ^Ab^	6.6 ± 0.3 ^Ab^
	B-NV	7.5 ± 0.1 ^Ba^	35.9 ± 0.1 ^Bb^	432.2 ± 0.2 ^Bb^	11.6 ± 0.2 ^Bb^
	N-PV	1.9 ± 0.2 ^Cb^	12.9 ± 0.1 ^Cb^	51.8 ± 1.6 ^Cb^	3.7 ± 0.1 ^Cb^
	B-PV	5.9 ± 0.1 ^Ab^	29.8 ± 0.1 ^Db^	323.9 ± 0.8 ^Db^	10.7 ± 0.3 ^Da^
	B-C1	82.9 ± 0.3 ^Db^	379.8 ± 0.3 ^Eb^	700.4 ± 0.1 ^Eb^	1.7 ± 0.1 ^Eb^
	B-C2	62.9 ± 0.1 ^Eb^	113.5 ± 0.3 ^Fb^	189.4 ± 0.4 ^Fb^	1.1 ± 0.1 ^Eb^

Different uppercase letters in the same column indicate significant differences (*p* < 0.05) among different treatments for the same storage time. Different lowercase letters indicate significant differences (*p* < 0.05) among different storage times for the same treatment, according to two-way ANOVA followed by Tukey’s HSD test.

**Table 2:** pH, titratable acidity (TA), moisture content and water activity (a_w_) evolution of non-enriched (N-NV and B-NV), pomegranate peel extract-enriched (N-PV and B-PV), and commercial buffered powders (B-C1 and B-C2) during standard storage at 22 °C for up to 6 months (mean (*n* = 3) ± standard deviation).

Time (Month)	Treatment	pH	TA (% *w*/*w*) *	Moisture Content (%)	a_w_
0	N-NV	3.4 ± 0.1 ^Aa^	8.2 ± 0.1 ^Aa^	0.9 ± 0.1 ^Aa^	0.47 ± 0.01 ^Aa^
	B-NV	6.5 ± 0.1 ^Ba^	0.4 ± 0.1 ^Ba^	0.8 ± 0.1 ^ACa^	0.18 ± 0.01 ^Ba^
	N-PV	3.8 ± 0.1 ^Cab^	5.8 ± 0.1 ^Ca^	0.5 ± 0.1 ^Ba^	0.26 ± 0.01 ^Ca^
	B-PV	6.3 ± 0.1 ^Da^	0.3 ± 0.1 ^Ba^	0.6 ± 0.1 ^BCab^	0.14 ± 0.01 ^Da^
	B-C1	5.9 ± 0.1 ^Eab^	3.6 ± 0.1 ^Da^	1.8 ± 0.1 ^Da^	0.26 ± 0.01 ^Ca^
	B-C2	6.2 ± 0.1 ^Da^	3.2 ± 0.4 ^Da^	0.5 ± 0.1 ^BCa^	0.30 ± 0.01 ^Ea^
1	N-NV	3.7 ± 0.1 ^Ab^	8.8 ± 0.8 ^Aa^	1.1 ± 0.1 ^Aa^	0.43 ± 0.01 ^Ab^
	B-NV	6.5 ± 0.1 ^Ba^	0.4 ± 0.1 ^Ba^	1.1 ± 0.2 ^Ab^	0.12 ± 0.01 ^Bb^
	N-PV	3.7 ± 0.1 ^Aa^	5.3 ± 0.3 ^Ca^	0.5 ± 0.1 ^Ba^	0.28 ± 0.01 ^Cb^
	B-PV	6.4 ± 0.1 ^Bab^	0.3 ± 0.1 ^Ba^	0.6 ± 0.1 ^Ba^	0.03 ± 0.01 ^Db^
	B-C1	5.8 ± 0.1 ^Ca^	2.7 ± 0.8 ^Da^	1.9 ± 0.1 ^Ca^	0.25 ± 0.01 ^Ea^
	B-C2	6.3 ± 0.3 ^Ba^	2.1 ± 0.3 ^Db^	0.9 ± 0.2 ^ABa^	0.29 ± 0.01 ^Ca^
3	N-NV	3.6 ± 0.1 ^Aab^	8.7 ± 0.7 ^Aa^	1.1 ± 0.1 ^Aa^	0.43 ± 0.01 ^Ab^
	B-NV	6.4 ± 0.1 ^Ba^	0.4 ± 0.1 ^Ba^	0.9 ± 0.1 ^Aab^	0.09 ± 0.01 ^Bc^
	N-PV	3.6 ± 0.1 ^Aa^	5.5 ± 0.5 ^Ca^	0.9 ± 0.2 ^Ab^	0.30 ± 0.01 ^Cc^
	B-PV	6.5 ± 0.1 ^Bb^	0.6 ± 0.1 ^Bb^	0.9 ± 0.1 ^Ab^	0.03 ± 0.01 ^Db^
	B-C1	6.1 ± 0.1 ^Cb^	2.6 ± 0.9 ^Da^	2.6 ± 0.6 ^Bab^	0.23 ± 0.01 ^Eb^
	B-C2	6.1 ± 0.1 ^Ca^	3.0 ± 0.1 ^Da^	3.4 ± 0.8 ^Bb^	0.26 ± 0.01 ^Fb^
6	N-NV	3.6 ± 0.1 ^Aab^	8.7 ± 0.3 ^Aa^	1.1 ± 0.1 ^Aa^	0.47 ± 0.02 ^Aa^
	B-NV	6.4 ± 0.1 ^Ba^	0.4 ± 0.1 ^Ba^	0.7 ± 0.1 ^Aa^	0.05 ± 0.01 ^Bd^
	N-PV	3.9 ± 0.1 ^Cb^	5.8 ± 0.5 ^Ca^	0.5 ± 0.1 ^Aa^	0.29 ± 0.01 ^Cd^
	B-PV	6.5 ± 0.1 ^Bb^	0.5 ± 0.1 ^Bc^	1.2 ± 0.1 ^Ac^	0.04 ± 0.01 ^Bb^
	B-C1	6.5 ± 0.1 ^Bc^	2.9 ± 0.6 ^Da^	3.0 ± 0.5 ^Bb^	0.21 ± 0.01 ^Dc^
	B-C2	6.3 ± 0.1 ^Ba^	2.5 ± 0.4 ^Dab^	3.9 ± 0.6 ^Cb^	0.22 ± 0.01 ^Dc^

* Values correspond to titratable acidity determined by NaOH titration and expressed as g acetic acid per 100 g of powder. Different uppercase letters in the same column indicate significant differences (*p* < 0.05) among different treatments for the same storage time. Different lowercase letters indicate significant differences (*p* < 0.05) among different storage times for the same treatment, according to two-way ANOVA followed by Tukey’s HSD test.

**Table 3:** Color values (L*, a*, b*), and total color differences (ΔE*) evolution of non-enriched (N-NV and B-NV), pomegranate peel extract-enriched (N-PV and B-PV), and commercial buffered powders (B-C1 and B-C2) during standard storage at 22 °C for up to 6 months (mean (*n* = 3) ± standard deviation).

Time (Month)	Treatment	L*	a*	b*	ΔE*
0	N-NV	98.0 ± 0.4 ^Aa^	−0.1 ± 0.1 ^Aab^	1.3 ± 0.1 ^Aa^	-
	B-NV	96.8 ± 0.8 ^Ba^	0.0 ± 0.1 ^Ba^	0.3 ± 0.1 ^Ba^	-
	N-PV	91.3 ± 0.1 ^Ca^	2.0 ± 0.1 ^Ca^	13.3 ± 0.1 ^Ca^	-
	B-PV	81.1 ± 0.5 ^Da^	1.7 ± 0.2 ^Da^	22.6 ± 0.3 ^Da^	-
	B-C1	95.2 ± 0.1 ^Ea^	−0.2 ± 0.1 ^Aab^	6.5 ± 0.1 ^Ea^	-
	B-C2	95.9 ± 0.1 ^BEa^	0.1 ± 0.1 ^Ba^	7.8 ± 0.1 ^Fa^	-
1	N-NV	98.2 ± 0.5 ^Aa^	−0.1 ± 0.1 ^Aa^	1.5 ± 0.1 ^Aab^	0.6 ± 0.1 ^Aa^
	B-NV	98.8 ± 0.4 ^Ab^	0.0 ± 0.1 ^Ab^	0.5 ± 0.1 ^Ba^	2.1 ± 0.5 ^BCa^
	N-PV	90.4 ± 0.4 ^Bb^	1.3 ± 0.1 ^Bb^	13.9 ± 0.1 ^Cb^	1.2 ± 0.3 ^ABa^
	B-PV	82.8 ± 0.1 ^Cb^	1.0 ± 0.1 ^Cb^	23.4 ± 0.1 ^Db^	2.0 ± 0.3 ^BCa^
	B-C1	94.1 ± 0.2 ^Dbc^	−0.3 ± 0.1 ^Da^	5.2 ± 0.3 ^Eb^	1.7 ± 0.4 ^ACa^
	B-C2	93.9 ± 0.5 ^Db^	−0.4 ± 0.1 ^Dbc^	6.4 ± 0.6 ^Fb^	2.5 ± 0.8 ^Ca^
3	N-NV	98.2 ± 0.3 ^Aa^	−0.1 ± 0.1 ^Aa^	1.8 ± 0.2 ^Ab^	0.7 ± 0.1 ^Aa^
	B-NV	98.8 ± 0.3 ^Ab^	0.0 ± 0.1 ^Aab^	0.6 ± 0.1 ^Bab^	2.0 ± 0.6 ^BDa^
	N-PV	90.6 ± 0.2 ^Bb^	2.4 ± 0.1 ^Bc^	14.6 ± 0.3 ^Cc^	1.5 ± 0.3 ^ABa^
	B-PV	84.4 ± 0.2 ^Cc^	0.6 ± 0.1 ^Cc^	26.2 ± 0.2 ^Dc^	4.9 ± 0.2 ^Cb^
	B-C1	94.5 ± 0.1 ^Dab^	−0.3 ± 0.1 ^Da^	4.7 ± 0.1 ^Eb^	1.9 ± 0.1 ^BDa^
	B-C2	93.7 ± 0.2 ^Db^	−0.5 ± 0.1 ^Eb^	6.5 ± 0.1 ^Fb^	2.6 ± 0.2 ^Da^
6	N-NV	97.8 ± 0.3 ^Aa^	−0.2 ± 0.1 ^Ab^	1.8 ± 0.1 ^Ab^	0.7 ± 0.2 ^Aa^
	B-NV	98.0 ± 0.8 ^Aab^	0.0 ± 0.1 ^Ba^	0.9 ± 0.3 ^Bb^	1.4 ± 0.5 ^ABa^
	N-PV	89.7 ± 0.1 ^Bc^	2.1 ± 0.1 ^Cd^	13.8 ± 0.1 ^Cb^	1.7 ± 0.1 ^Ba^
	B-PV	83.9 ± 0.1 ^Cc^	0.7 ± 0.1 ^Dc^	24.1 ± 0.1 ^Dd^	3.3 ± 0.3 ^Cc^
	B-C1	93.6 ± 0.5 ^Dc^	−0.2 ± 0.1 ^ABb^	7.5 ± 0.1 ^Ec^	1.9 ± 0.4 ^Ba^
	B-C2	92.5 ± 0.3 ^Dc^	−0.3 ± 0.2 ^Ac^	8.0 ± 0.1 ^Fa^	3.4 ± 0.4 ^Ca^

Different uppercase letters in the same column indicate significant differences (*p* < 0.05) among different treatments for the same storage time. Different lowercase letters indicate significant differences (*p* < 0.05) among different storage times for the same treatment, according to two-way ANOVA followed by Tukey’s HSD test.

**Table 4:** TPC (mg GAE/g_powder_) and TAC (µmol TE/g_powder_) of non-enriched (N-NV and B-NV), pomegranate peel extract-enriched (N-PV and B-PV), and commercial buffered powders (B-C1 and B-C2) during standard storage at 22 °C for up to 6 months (mean (*n* = 3) ± standard deviation).

Time (Month)	Treatment	TPC * (mg GAE/g_powder_)	TAC * (µmol TE/g_powder_)
0	N-NV	0.3 ± 0.1 ^Aa^	4.0 ± 0.1 ^Aa^
	B-NV	0.1 ± 0.1 ^Ba^	2.4 ± 0.6 ^Ba^
	N-PV	27.0 ± 3.9 ^Ca^	287.8 ± 5.6 ^Ca^
	B-PV	4.1 ± 0.2 ^Da^	38.0 ± 1.1 ^Da^
	B-C1	0.1 ± 0.1 ^Ba^	3.3 ± 0.5 ^ABa^
	B-C2	0.1 ± 0.1 ^Ba^	2.7 ± 0.3 ^Ba^
1	N-NV	0.3 ± 0.1 ^Aa^	3.6 ± 0.3 ^Aa^
	B-NV	0.1 ± 0.1 ^Ba^	2.3 ± 0.3 ^Ba^
	N-PV	27.3 ± 0.6 ^Ca^	282.8 ± 2.7 ^Cab^
	B-PV	4.6 ± 0.3 ^Dac^	38.7 ± 0.8 ^Da^
	B-C1	0.1 ± 0.1 ^Ba^	3.1 ± 0.7 ^ABa^
	B-C2	0.1 ± 0.1 ^Ba^	2.6 ± 0.5 ^ABa^
3	N-NV	0.2 ± 0.1 ^Aa^	4.1 ± 0.3 ^Aa^
	B-NV	0.1 ± 0.1 ^Ba^	2.5 ± 0.5 ^Ba^
	N-PV	27.8 ± 2.0 ^Ca^	275.1 ± 3.5 ^Cb^
	B-PV	6.0 ± 0.5 ^Db^	37.5 ± 0.5 ^Da^
	B-C1	0.1 ± 0.1 ^ABa^	3.5 ± 0.4 ^ABa^
	B-C2	0.1 ± 0.1 ^Ba^	2.9 ± 0.6 ^Ba^
6	N-NV	0.3 ± 0.1 ^Aa^	3.6 ± 0.4 ^Aa^
	B-NV	0.1 ± 0.1 ^Ba^	2.5 ± 0.4 ^Ba^
	N-PV	27.9 ± 1.5 ^Ca^	279.3 ± 1.7 ^Cab^
	B-PV	5.3 ± 0.4 ^Dbc^	37.1 ± 0.9 ^Da^
	B-C1	0.1 ± 0.1 ^Ba^	2.4 ± 0.4 ^Ba^
	B-C2	0.1 ± 0.1 ^Ba^	3.5 ± 0.2 ^Aa^

* Results are expressed per gram of spray-dried powder. Different uppercase letters in the same column indicate significant differences (*p* < 0.05) among different treatments for the same storage time. Different lowercase letters indicate significant differences (*p* < 0.05) among different storage times for the same treatment, according to two-way ANOVA followed by Tukey’s HSD test.

**Table 5:** Inhibition zone diameter (mm) for non-enriched (N-NV and B-NV), pomegranate peel extract-enriched (N-PV and B-PV), and commercial buffered powders (B-C1 and B-C2) during standard storage at 22 °C for up to 6 months against *Listeria monocytogenes* STCC 4032 and *Salmonella enterica* STCC 443 (mean (*n* = 3) ± standard deviation).

Time (Month)	Treatment	Inhibition Zone Diameter (mm)	
		*L. monocytogenes* STCC 4032	*S. enterica* STCC 443
0	N-NV	29.6 ± 4.0 ^ACa^	18.9 ± 2.0 ^Aa^
	B-NV	9.5 ± 0.2 ^Ba^	NA ^Ba^
	N-PV	26.5 ± 0.8 ^ACa^	15.5 ± 1.3 ^Aa^
	B-PV	24.9 ± 0.6 ^Ca^	NA ^Ba^
	B-C1	11.3 ± 1.2 ^Ba^	NA ^Ba^
	B-C2	7.5 ± 0.5 ^Ba^	NA ^Ba^
	Oxytetracycline dihydrate, 100 mg/L	31.4 ± 1.9 ^Aa^	29.5 ± 2.2 ^Ca^
6	N-NV	28.4 ± 2.5 ^ACa^	18.4 ± 0.7 ^Aa^
	B-NV	8.7 ± 0.5 ^BEa^	NA ^Ba^
	N-PV	26.1 ± 1.1 ^CDa^	15.6 ± 0.4 ^Ca^
	B-PV	24.6 ± 0.6 ^Da^	NA ^Ba^
	B-C1	11.3 ± 0.5 ^Ba^	NA ^Ba^
	B-C2	7.3 ± 0.2 ^Ea^	NA ^Ba^
	Oxytetracycline dihydrate, 100 mg/L	31.5 ± 0.9 ^Aa^	30.4 ± 0.5 ^Da^

Different uppercase letters in the same column indicate significant differences (*p* < 0.05) among different treatments for the same storage time. Different lowercase letters indicate significant differences (*p* < 0.05) among different storage times for the same treatment, according to two-way ANOVA followed by Tukey’s HSD test. NA = no measurable activity observed. Oxytetracycline dihydrate (100 mg/L) was used as a positive control. Negative control (sterilized distilled water) did not show any activity.

**Table 6:** Identification of the experimental and commercial spray-dried powder formulations according to the presence of pomegranate peel extract (non-enriched/enriched) and the buffering treatment applied (non-buffered/buffered).

Plant-Derived Extract	Formulation	Buffering State	Nomenclature
Absent	❖ Non-enriched	Non-buffered	N-NV
Absent	❖ Non-enriched	Buffered	B-NV
			
Present	❖ Pomegranate peel extract-enriched	Non-buffered	N-PV
Present	❖ Pomegranate peel extract-enriched	Buffered	B-PV
			
Commercial reference	❖ Verdad^®^ Powder N6	Buffered	B-C1
Commercial reference	❖ BactoCEASE™ NV DRY (EU)	Buffered	B-C2

## Data Availability

The original contributions presented in the study are included in the article. Further inquiries can be directed to the corresponding author.
